# Development of a computational model to inform environmental surveillance sampling plans for *Salmonella enterica* serovar Typhi in wastewater

**DOI:** 10.1371/journal.pntd.0011468

**Published:** 2024-03-29

**Authors:** Elisabeth Burnor, Cory W. Morin, Jeffry H. Shirai, Nicolette A. Zhou, John Scott Meschke

**Affiliations:** Department of Environmental and Occupational Health Sciences, University of Washington School of Public Health, Seattle, Washington, United States of America; Institute for Health Metrics and Evaluation, UNITED STATES

## Abstract

Typhoid fever–an acute febrile disease caused by infection with the bacterium *Salmonella enterica* serotype Typhi (*S*. Typhi)—continues to be a leading cause of global morbidity and mortality, particularly in developing countries with limited access to safe drinking water and adequate sanitation. Environmental surveillance, the process of detecting and enumerating disease-causing agents in wastewater, is a useful tool to monitor the circulation of typhoid fever in endemic regions. The design of environmental surveillance sampling plans and the interpretation of sampling results is complicated by a high degree of uncertainty and variability in factors that affect the final measured pathogens in wastewater samples, such as pathogen travel time through a wastewater network, pathogen dilution, decay and degradation, and laboratory processing methods. Computational models can, to an extent, assist in the design of sampling plans and aid in the evaluation of how different contributing factors affect sampling results. This study presents a computational model combining dynamic and probabilistic modeling techniques to estimate–on a spatial and temporal scale–the approximate probability of detecting *S*. Typhi within a wastewater system. This model may be utilized to inform environmental surveillance sampling plans and may provide useful insight into selecting appropriate sampling locations and times and interpreting results. A simulated applied modeling scenario is presented to demonstrate the model’s functionality for aiding an environmental surveillance study in a typhoid-endemic community.

## Author summary

Millions of cases of typhoid fever occur each year, causing thousands of deaths, particularly in children. In order to distribute vaccines to communities with the highest need, it is important to understand disease prevalence in different regions. Clinical surveillance for typhoid fever requires testing of blood and fecal samples, which can be challenging, especially in communities that do not have easy access to healthcare. Environmental surveillance is another method of disease surveillance where wastewater samples are tested for the bacteria that causes typhoid fever–*Salmonella* Typhi. Environmental surveillance allows researchers to infer information about the number of people who may be infected in a community by measuring the number of bacteria in wastewater samples. Researchers must determine when and where to collect wastewater samples in a complex wastewater network, and the interpretation of this data is complicated. Many different variables affect the concentration of bacteria in wastewater, including how many bacteria are shed in the stool of people with infections and how much the bacteria is diluted and degraded before it is measured. This study describes a model developed to aid with the design of an environmental sampling plan for typhoid fever and to help with the interpretation of results.

## 1. Introduction

Typhoid fever is an enteric infectious disease caused by the etiologic agents *Salmonella enterica* serovars Typhi and Paratyphi (*S*. Typhi and *S*. Paratyphi) [1]. Patients with typhoid fever commonly experience prolonged fever, headaches, and abdominal pain [2]. As of 2017, the burden of typhoid fever in low and middle-income countries (LMICs) was estimated to be 17.8 million cases per year, with highest incidence among children aged 2–4 years old [3]. In 2018 and 2020, the World Health Organization prequalified two typhoid conjugate vaccines (TCV) and has adopted the use of TCVs in parts of the world with a high burden of typhoid [4–6]. Countries considering how and where to provide TCVs need accurate and geographically representative information about typhoid incidence in urban and rural areas [7]. However, the true burden of typhoid is difficult to determine, and data on the incidence of typhoid fever in LMICs remain sparse [3]. Active and passive clinical typhoid fever surveillance relies on clinical-specimen culture surveillance, which requires the detection of pathogens in blood, bone marrow, stool or urine of individual patients [2]. This type of surveillance is resource-intensive, requires large numbers of participants, and depends on robust laboratory and medical infrastructure [4].

Environmental surveillance (ES) has been used to monitor the circulation of multiple pathogens, including poliovirus, measles, enteroviruses, hepatitis A, hepatitis E, norovirus, SARS-CoV-2, and others [8]. The COVID-19 pandemic, in particular, brought ES to the attention of the global academic and public health community, and SARS-CoV-2 ES has been adopted as a complementary surveillance method to clinical testing for COVID-19 in hundreds of countries [8,9]. ES is currently being implemented to monitor the transmission of typhoid fever in some endemic geographic areas in order to overcome gaps in clinical population-based surveillance in LMICs [4,10,11]. ES offers an anonymous, non-invasive approach to monitor pathogen circulation in a population that does not rely on clinical testing availability and behavior and can be less expensive than many other forms of clinical surveillance [4,8]. Additionally, some individuals infected with *S*. Typhi are likely to be missed by clinical surveillance methodologies, as some proportion of infected individuals are known to become asymptomatic, chronic carriers, capable of shedding large numbers of bacteria into the environment, but unlikely to seek medical care or testing [12–14]. Because ES is capable of monitoring for the presence of a pathogen within parts of a community served by a sewer network, regardless of clinical testing availability or utilization, ES for typhoid can provide an ideal complementary method to clinical surveillance for monitoring typhoid transmission and prevalence.

When developing an ES sampling plan, the identification of optimal sampling locations and sampling times that maximize the probability of detection while being logistically feasible presents a challenge [15]. Once an ES study has begun and sampling data are analyzed, the interpretation of positive and negative results is complicated by a high degree of uncertainty as to how the measured pathogen concentrations relates to the number of infections in a catchment area [16,17]. Additionally, laboratory methods for the detection and quantification of pathogens in wastewater vary, and there is a lack of standardization in methodologies, further complicating the interpretation of ES results [18,19]. The sensitivity of an ES sampling plan to detect and quantify pathogens and produce data interpretable for public health use is limited by the volume of wastewater sampled and the sensitivity of the applied laboratory methods [20].

These challenges involved in designing an optimal ES study can be partially addressed through computational modeling or site selection algorithms, and previous studies have proposed different approaches. As part of efforts to design sampling plans for SARS-CoV-2 ES, a number of studies have proposed modeling methods for optimal sampling site selection based on spatial sewer network maps [21–24]. These models aim to maximize the population covered by ES while minimizing the number of sampling sites required to identify circulation of SAR-CoV-2 in the overall community and to pinpoint disease hotspots within sub-catchment areas. These studies did not model pathogen fate and transport in wastewater networks or disease transmission dynamics but focused on the distribution of catchment populations and manholes, the cumulative service areas within sewer networks, and the effective monitoring areas covered by each manhole. Similarly, a study on SARS-CoV-2 sampling by Wang et al., (2023) utilized a network map of sewage lines to design a sampling plan, and then, once sampling began, applied an adaptive sampling process to modify sampling locations in response to negative and positive results from multiple rounds of sampling, with the ultimate goal of identifying COVID-19 hotspots [25].

Other environmental sampling models have incorporated disease transmission dynamics and pathogen fate, transport, and decay. Ranta et al. (2001) simulated the probability of detection of poliovirus at a single sampling location at the convergence of a centralized piped wastewater network, given different modeled transmission scenarios, sampling schemes, and laboratory techniques in order to understand the sensitivity of environmental surveillance in different scenarios [20]. A more recently published model was developed to optimize an environmental sampling plan for typhoid fever in a piped sewer network in Kolkata, India, with unknown sewerage structure [26]. This study modeled shedding rates, pathogen fate, transport, and decay, and laboratory method sensitivity to simulate different typhoid transmission and sampling scenarios in Kolkata. The authors then applied an adaptive method for ES sampling site allocation to improve the likelihood of detection of *S*. typhi, if it is circulating anywhere in the community. Similar to Wang et al., (2023), the adaptive sampling algorithm optimizes sampling site locations using multiple rounds of sampling results [25,26]. All of these previously described models were implemented in formal, centralized, engineered wastewater networks with underground pipes.

The primary aim of the model presented in this study is to inform sampling plans in study locations that are not served by formal, piped, centralized wastewater networks, but by open drainage networks, urban freshwater rivers and streams, or a combination of both open channel flow and piped systems. Centralized, piped sewer networks have minimum flow volume and velocity requirements that prevent pipe blockages and biofilm formation, allowing previous modeling studies to make reasonable, simplifying assumptions about the residence times of pathogens in a wastewater network (e.g. less than one day) [26,27]. In a wastewater network with open channels or environmental streams, flow dynamics and the movement of pathogens through the system are likely to be much more variable. This model also focuses on the effects of flow dilution and decay of pathogens as they travel downstream and incorporates diurnal variability wastewater flow and fecal shedding over the course of a 24-hour day.

This study presents a flexible, dynamic computational model that is highly tunable to specific ES study areas and designed to be broadly applicable in communities with informal wastewater drainage networks. Given simulated typhoid prevalence, shedding rates, wastewater flow, environmental flow, pathogen loading, pathogen fate and transport, sampling volume, and laboratory method sensitivity, this model produces estimates of the probability of detection along the wastewater network and throughout a 24-hour day. The output from this model may be used to aid in the design of an environmental sampling plan, inform decisions about sampling locations and times, and aid in the interpretation of results from an environmental surveillance program. We present a theoretical application of this model within a community in part of Vellore City, India.

## 2. Methods

### 2.1 Mapping

**Fig 1:**
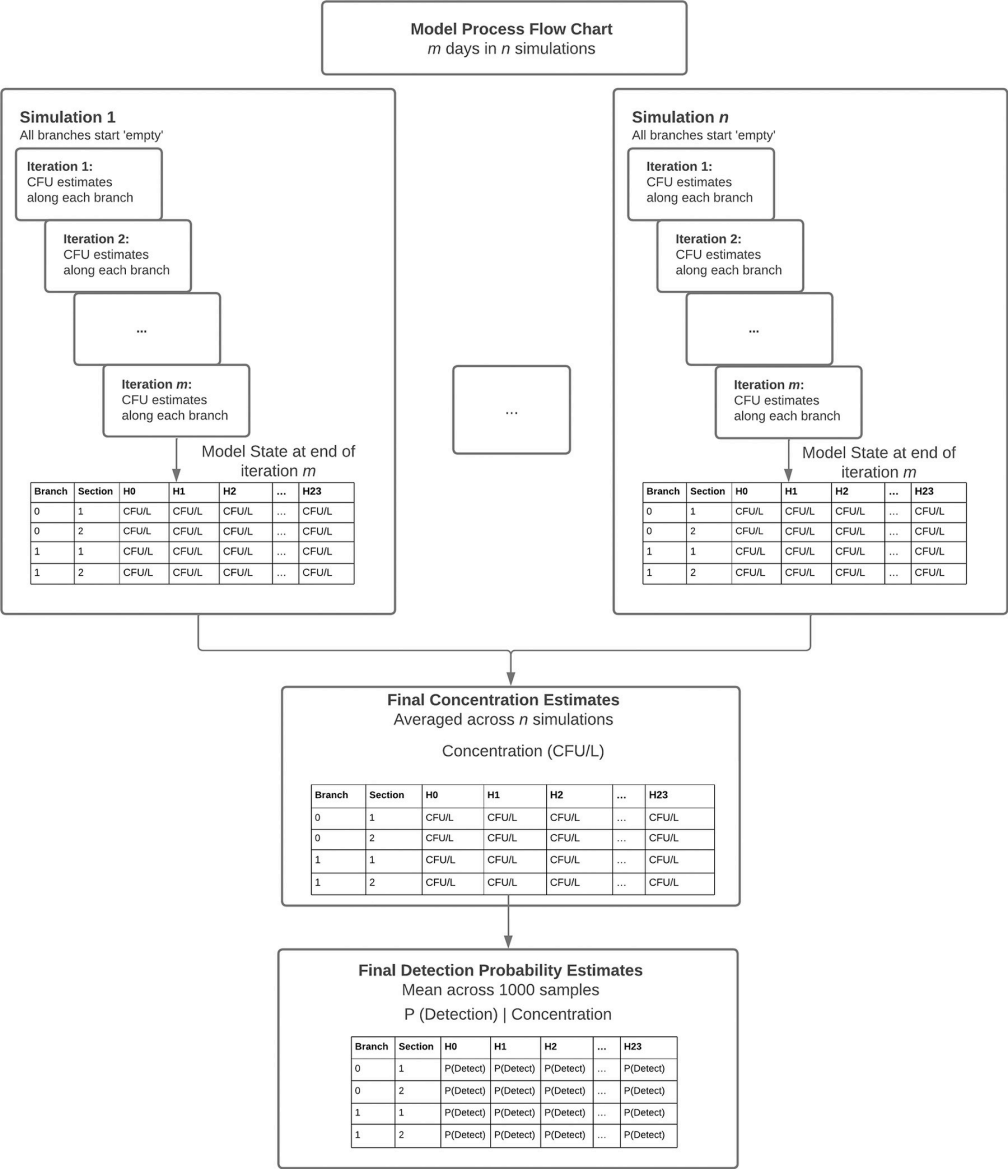
Drainage map of the sewage network of part of Vellore City, India. This map was utilized in this applied scenario and identifies the organization and IDs of major drainage branches. This map is used to define the fixed parameters (Table 1), which identify the length of each branch and each branch’s parent branch(es) in order to represent the direction of wastewater flow. This map provides a visualization of the conceptual map used by the model to track wastewater flow along the sewage network. Blue arrows indicate the direction of flow toward the convergence point. Note: not all branch IDs are shown to improve readability. Drainage map from https://es.world/map/vellore for drainage lines [29]. Street map from OpenStreetMap contributors. (2024) [Data file from 2023-06-02] Retrieved from https://www.openstreetmap.org/export—map=13/12.9049/79.1300 [43]. Terms: https://www.openstreetmap.org/copyright.

In order to run this model, a simple geospatial vector map of the wastewater or drainage network of the community in question is required. Some communities lack centralized wastewater management systems and associated maps [10,28]. For areas where piped wastewater systems or mapped drainage networks are not available or where wastewater is discharged into open channels and environmental streams, a company called Novel-T has produced an environmental surveillance mapping service (es.world), which provides geospatial maps of synthetic streams and waterways, based on local topology [29]. These maps are designed to identify ES sampling sites in streams or drainage systems and associated catchment areas. The simulation provided in this paper utilizes a theoretical drainage map shapefile of Vellore City, India, created by Novel-T. The input parameters for Table 1 were prepared by GIS analysis of this shapefile in R. Briefly, the ‘convergence point’ (the ‘end’ of the system where all drainage lines feed) of the system was identified (see Fig 1). From there, a map was created of each branch in the system, totaling 66 branches, and the geospatial coordinates of the start point and end point of each branch were used to determine the parent branches feeding into each branch. This information was incorporated into a table of model input parameters, in which each row defines a single branch, the branch’s parent(s), the length of the branch, the branch’s estimated population, and that population’s theoretical disease prevalence (see 2.3 Model Parameterization and Table 1). The shapefiles available on es.world include population estimates along each drainage line, which are estimated by overlaying grid population raster data (at a resolution of approximately 100 meters) along drainage lines within catchment areas [30–32]. Population is assigned to each branch according to the population raster. For this model application, these population estimates generated by Novel-T were used [29].

**Table 1:** Model Parameters for theoretical applied scenario.

Model Input	Type (Fixed or Distributed)^1^	Distribution and Parameter Values	Units	Model Application Inputs^2^
Branch ID	Fixed	Numerical ID		IDs assigned
Parent Branches	Fixed	Numerical IDs for each parent or -1 (for no parent)		Estimated from DEM models (es.world)
Velocity	Calculated from flow and cross sectional branch area	Calculated proportional to flow V = Q / A	meters/hour	Sensitivity variable
Cross sectional branch area	Fixed	1 meter x 0.5 meters	m^2	Assumption
Branch Length	Fixed	Estimated by GIS analysis	m	Estimate from geospatial analysis/DEM models [29]
Branch Population	Fixed	Estimated from population raster overlay (GIS analysis)	persons	Estimated from es.world [29–32]
Prevalence	Fixed	Low: 2 Medium: 20 High: 200	cases/100,000 persons	Sensitivity variable [14]
Given infection, day of infection	Distribution	Randomly sample from day 0 to day 14, with equal weights	days since infection	[33]
Probability of Shedding, given day of infection	Assigned based on day of infection	Event probability (0 to 1)		[33]
Typhoid Shedding Rate (given shedding)	Distribution	Lognormal (μ = 11.9, σ = 0.228)	CFU/gram feces	Expert opinion
Fecal Output	Distribution	N(μ = 243, σ = 130)	feces/person-day	[34]
Wastewater Flow	Distribution	N(μ = 100, σ = 50)	liters/person-day	[35]
Environmental Flow	Distribution	Very low: N(μ = 300, σ = 10) Low: N(μ = 3,000, σ = 100) Medium: N(μ = 30,000, σ = 1,000) High: N(μ = 300,000, σ = 10,000)	liters/hour	Sensitivity Variable. [36]
Decay Rate	Distribution	Random sample from two point estimates, with equal weights λ in (0.014, 0.043); N(t) = N0 * e ^(λt)		[37,38]
Wastewater Flow over 24 hour period	Fixed	Proportion of total daily flow occurring at each hour in 24-hour cycle		[39–41]
Fecal Output over 24-hour period	Fixed	Proportion of total daily fecal output occurring at each hour in a 24-hour cycle		[42]

^1^ Fixed inputs are held constant for each model simulation, but can be adjusted to compare output across different disease and flow scenarios. Distributed inputs are defined as distributions and these distributions are randomly sampled for each person along a branch (fecal output, probability of shedding, and shedding rate), for each branch (wastewater flow and environmental flow), and for the entire system (decay rate) at the beginning of each simulation in order to integrate the natural variability inherent in each of these parameters into the model’s total uncertainty. The shapes and parameters of each distribution may be adjusted between model runs to compare outputs across different flow scenarios, different assumptions about shedding, etcetera.

^2^ Details of all parameter inputs used in the model application presented here can be found in S1 Text.

### 2.2 Code

The GIS analysis of the drainage map was performed using R v. 4.1.0 [44]. The dynamic computational model was implemented in Python v. 3.7.0 [45]. All code is publicly available.

### 2.3 Model parameterization

Table 1 summarizes the input parameters required for the dynamic model and indicates whether the parameter is fixed throughout all model simulations or randomly sampled at the start of each simulation. These parameters describe each branch of a drainage network and how each branch in the network interacts with upstream and downstream branches by assigning Branch IDs and Branch Parent IDs. For this study, shapefiles for theoretical drainage lines in parts of Vellore City, India were adapted as described in Methods 2.1 to produce a system map defining the Branch IDs, Parent Branches, Branch Lengths, and Branch Populations in Table 1 [29].

If no maps or shapefiles exist for a community, it is possible to produce a system map and inputs for Table 1 via analysis of open-source satellite imagery of street maps in order to create a theoretical map of community wastewater networks, which commonly coincide with road infrastructure [22]. A manually prepared map using road networks would require an estimate of flow direction. Where mapped wastewater lines and theoretical drainage lines are not available, a manual mapping approach would be time consuming and is likely be more feasible in small communities with few drainage lines.

Estimates and literature sources for the parameters of this model application in Vellore City, India are indicated in Table 1 and more detailed explanations for input parameters and data sources are described in S1 Text. Any of these parameters may be adjusted. Estimates for environmental (or background) flow volume may be measured directly at a few locations within the wastewater drainage system using flow meters, hydrological modeling, velocimeters, or other measurement methodologies for calculating flow volume [46]. These measurements may then be used to create an environmental flow distribution for the system or may be used to assign individual flow distributions by branch. Environmental flow is included as a distributed parameter and is randomly sampled at the beginning of each simulation for each branch from the pre-defined distribution. Wastewater flow per capita per day is likely to vary by community and region and should be estimated based on locally relevant data or community surveys [35,36,47]. Given environmental flow, wastewater flow, and an assumed cross-sectional area for each branch, the velocity for each branch is calculated in proportion to average total estimated flow of that branch at the beginning of each simulation (Eq 1)

$$velocity\;=\;\frac{Flow}{Cross‐sectinalArea}$$ Eq 1

Wastewater flow into the system and defecation rates are variable throughout a 24-hour daily cycle, so the model is designed to update every hour according to the estimated wastewater flow and defecation rates at each hour of the day. For this model application, estimates of diurnal variation in wastewater flow and defecation were obtained from available literature and are described in detail in S1 Text. Wastewater use and disposal throughout the day and defecation rates are likely to vary by region and population, and model accuracy would be improved by obtaining local information about these patterns through household surveys or water meter data, as has previously been done in applied modeling studies [47].

Point prevalence of typhoid infections (whether symptomatic or asymptomatic) are assigned by branch. Prevalence may be assigned uniformly throughout the system or it may vary by branch in order to simulate case clustering in neighborhoods or larger areas (in this study, prevalence is assigned uniformly). At the start of each model simulation, each person living along a branch is randomly assigned as ‘infected’ or ‘not infected’, according to each branch’s assigned prevalence. For each person assigned as ‘infected’, the number of days since the initial infection is randomly selected from a uniform distribution ranging from 0 to 14, and the probability of intermittent shedding is assigned according to the number of days since infection [33] (Fig A in S1 Text). The person is randomly assigned to be shedding or not shedding, based on the intermittent shedding probability. If the person is shedding, the number of *S*. Typhi bacteria shed per gram of feces is randomly sampled from a lognormal distribution, with parameters described in Table 1 and in Fig B in S1 Text. Daily defecation rates are randomly sampled from a normal distribution (Table 1), and the total number of *S*. Typhi bacteria shed per shedding person per day is calculated along each branch (see S1 Text and S2 Text for more details).

### 2.4 Initialization

**Fig 2:**
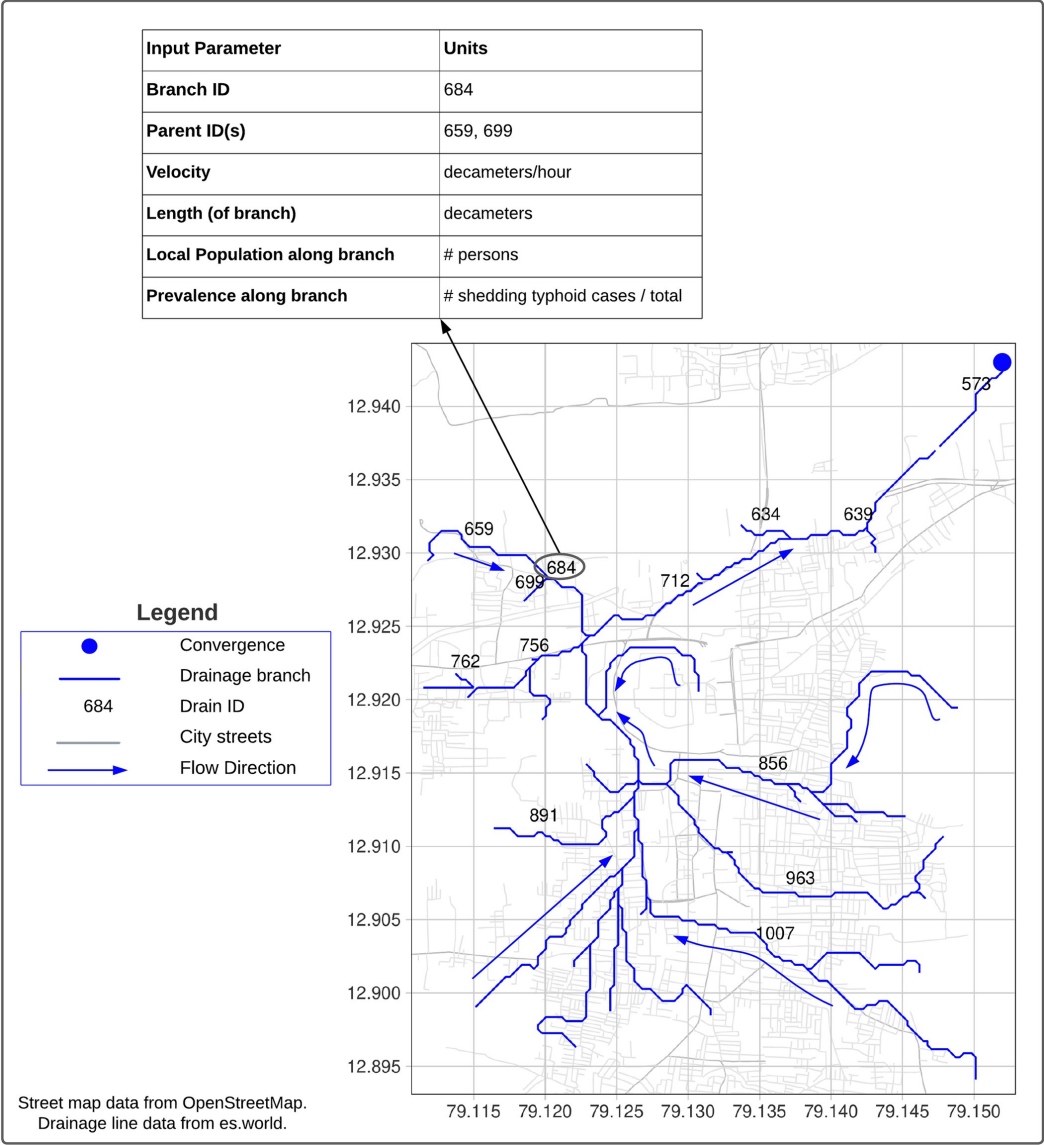
Diagram of the model processing steps. Each simulation box represents one of *n* simulations, for which values for distributed parameters are randomly selected from parameter distributions. Within each simulation, the model iterates over *m* iterations and stores calculations for pathogen concentrations and detection probability at the end of day *m*. Once *n* simulations are completed, the model computes averages for pathogen concentrations across all simulations (and associated standard error and confidence intervals). The model then computes average detection probabilities (and associated standard error and confidence intervals as described in 2.6 Probability of Detection and in S2 Text).

Once the model has been parameterized and a theoretical environmental system map is defined, the model will run for *m* iterations within *n* simulations. Fig 2 presents a flowchart demonstrating the mechanics of the model. At the beginning of each simulation, the model system is initialized with parameter data (e.g. the data presented in Table 1). All model inputs labeled as “Distributed” in Table 1 are randomly sampled from the input’s defined distribution at the beginning of each simulation. After these parameters have been randomly sampled, they then remain fixed for all iterations within the simulation. Model inputs labeled as “Fixed” are held constant over all model simulations and all iterations. When the model is initialized for a simulation, it will run for multiple iterations within the simulation (where each iteration represents a 24-hour cycle). Multiple iterations are included within each simulation because the system starts from empty, where all branches contain no wastewater flow volume and zero pathogens. During each iteration, flow- and pathogen-loading occur along each branch and the downstream movement of both are tracked over the 24-hour cycle. Each subsequent iteration includes data from the previous iteration (i.e., if a branch of the network has x pathogens at 23:00 in the first iteration, those pathogens will be incorporated into downstream branches at 00:00 of the following iteration). The iterations end when the model reaches steady-state, defined here as a mean absolute difference across all branches and all hours of less than 0.1 pathogens per liter (10 fold lower than the lowest seeding concentration evaluated in Zhou et al., 2023 [48]). When the model has run for *m* iterations, a simulation is complete, and all distributed parameters are resampled for the next simulation.

This model does not simulate changes in shedding or transmission dynamics over the course of multiple days, but produces point estimates of pathogen concentrations and detection probabilities throughout the wastewater network, given an assumed point prevalence of typhoid shedders. In real multi-day or multi-week transmission scenarios, and in particular, in outbreak scenarios such as those modeled in Ranta (2001), the number of new infections and new shedders in the environment, the number if infected people who are intermittently shedding, and the number of recovered persons who are no longer shedding will change over time [20]. Although simulating an evolving transmission and fluctuating shedding scenario in conjunction with this model would be an useful next step, this is beyond the scope of the current study.

### 2.5 Branch updates

At every hour, the model starts at the convergence, or ‘end’ of the system (see the ‘Convergence’ point in Fig 1) and calculates the number of pathogens and total flow volume (including wastewater flow volume and environmental volume) for each section along each branch. The model follows each branch from the convergence outward by identifying parent branches and continuing to move from child to parent branch until it reaches a branch that does not have any parent branches (for example, Branches 659 and 891 in Fig 1). At the start of each hour, given estimated flow velocity in each branch, the model calculates how far a compartment of water volume and diluted pathogens have traveled in one hour. If this distance is less than one section, then the wastewater flow input and pathogen input for that section are estimated for the population along that section of the branch (Fig A in S2 Text) and combined with environmental flow. If the distance travelled is longer than one section, then the total flow volume and pathogens from upstream sections are added to the section’s flow and pathogen inputs. If the distance travelled is longer than the entire branch, the model calculates flow and pathogen input from parent branches (Fig A in S2 Text). As a result, for every hour and for each section of each branch, the total flow and pathogen estimates are a combination of upstream inputs from the previous hour that have flowed downstream over time, in addition to the flow and pathogens that have entered the system over the course of the current hour. Further details on the equations defining pathogen and wastewater flow loading can be found in S2 Text.

### 2.6 Probability of detection

Once the model has completed *m* iterations and *n* simulation, the estimated detection probability at each section along each branch of the system for each hour of the day is calculated. The detection probability is dependent on the sensitivity of the laboratory method being modeled and the volume of wastewater sampled (S2 Text). Probability of detection is calculated according to the formula described in Ranta, 2001 [20]. The calculation of the detection probability incorporates sampling variability by randomly sampling from a Poisson distribution where the distribution parameter (λ) is equal to the mean pathogen concentration in that section and hour across all *n* simulations. This calculation incorporates the sensitivity of the laboratory method and the volume of wastewater being sampled, and both can be adjusted to model different sampling volumes and different method sensitivities (See Equations 6, 7, and 8 in S2 Text). The mean, standard deviation, and 95% confidence intervals are calculated for the estimate of detection probability for each section and hour. (See S2 Text for further details.)

### 2.7 Uncertainty

Uncertainty in model estimates is incorporated by running the model for 1,000 simulations, as described in Methods 2.4 and Fig 2, and resampling distributed parameters for each simulation. For each final estimate of pathogen concentration throughout the wastewater network, the mean concentration, standard deviation, and 95% confidence intervals are computed across model simulations. For each estimate of detection probability, the final estimates of detection probabilities with associated 95% confidence intervals incorporate the natural sampling variability inherent in any environmental sampling methodology (dependent on sample volume and the random distribution of pathogens captured within a single sample), the population variability inherent in many of the model input parameters (such as daily fecal output, wastewater flow production, and shedding rates), and uncertainty in model input parameters, such as environmental flow and pathogen decay rate (Table 1).

### 2.8 Applied scenario

In order to demonstrate the modeling framework’s functionality, an applied scenario was performed in an approximation of the wastewater drainage network in part of Vellore City, India. In parts of Vellore City, India, an artificial open above-ground shallow drainage network conveys wastewater through the system [10]. This model was conceptualized to aid in the development of an environmental surveillance sampling plan for non-centralized, non-piped wastewater drainage networks, or for systems that include a combination of open drains, environmental streams, pipes, pump stations, and treatment plants. Given this, Vellore City is a reasonable location to apply the model. Although this model could be adapted for a formal, centralized wastewater network, it is likely to be more useful for systems that do not have consistent minimum flow rates throughout each day and between seasons.

### 2.9 Sensitivity analysis

Twelve scenarios were examined with varying model input parameters for typhoid prevalence and environmental flow (i.e., background daily flow through the system from sources other than household wastewater contributions). Accurate community prevalence estimates are difficult to ascertain without the infrastructure needed for active or passive clinical surveillance programs. Depending on the region where an ES study is being conducted, study partners may not have detailed knowledge of local prevalence. Despite this knowledge gap, an assumption of point prevalence within a study area is required to model the shedding, fate, transport, decay, and dilution of *S*. Typhi through a system. In order to examine a range of possible estimates of infection and shedding prevalence in a community, three point prevalence estimates were included. The ‘Medium’ prevalence in this sensitivity analysis corresponds to a point prevalence estimated from a recently published active clinical surveillance study measuring typhoid incidence in children under 15 years old [14]. The ‘Low’ and ‘High’ prevalence rates correspond to point prevalence estimates ten times lower and ten times higher than this clinical surveillance-based estimate, respectively (Table 2). In these scenarios, prevalence estimates were assigned uniformly to all branches, although the model structure allows for prevalence to vary by branch.

**Table 2:** Model Inputs for Sensitivity Analyses.

Prevalence		Environmental Flow	
Scenario	Cases/100,000	Scenario	Liters per hour (per channel branch section) Mean (SD)
Low	2	Very Low	300 (10)
	2	Low	3,000 (100)
	2	Medium	30,000 (1,000)
	2	High	300,000 (10,000)
Medium	20	Very Low	300 (10)
	20	Low	3,000 (100)
	20	Medium	30,000 (1,000)
	20	High	300,000 (10,000)
High	200	Very Low	300 (10)
	200	Low	3,000 (100)
	200	Medium	30,000 (1,000)
	200	High	300,000 (10,000)

Given uncertainty about background flow in open channels in this study area and known seasonal fluctuations in rainfall in this region of southern India [49], four flow scenarios were examined at each level of prevalence in order to examine the effects of a broad range of environmental flow rates on model output. (S1 Text).

## 3. Results

### 3.1 Model scenario example

A ‘base’ case of medium point prevalence of *S*. Typhi shedding (20 cases/100,000 person) and low environmental flow is used to demonstrate how model output may be analyzed and interpreted. For this model run, a spin-up time of three iterations resulted in steady-state model output, and the model was run for 1,000 simulations (runtime of 23 minutes). A resolution of 10 meters was used for branch sections. A detection probability of 83% at a pathogen concentration of one CFU per liter wastewater and a sampling volume of six liters were used as the laboratory parameters for this applied scenario (Equations 6 and 7 in S2 Text). The laboratory sampling volume was based on the 2-inch filter cartridge (FC1-D) method described in Zhou et al. 2023, which measures six liters of wastewater at time [48]. As described in Fig 2, upon completion of all model simulations, tables are produced with the average *S*. Typhi concentrations and the average detection probabilities for each 10-meter section of each branch in the wastewater network, over the course of a 24-hour day (tables with standard deviations for both concentration and detection probability are also produced).

### 3.2 Model run output and interpretation

**Fig 3:**
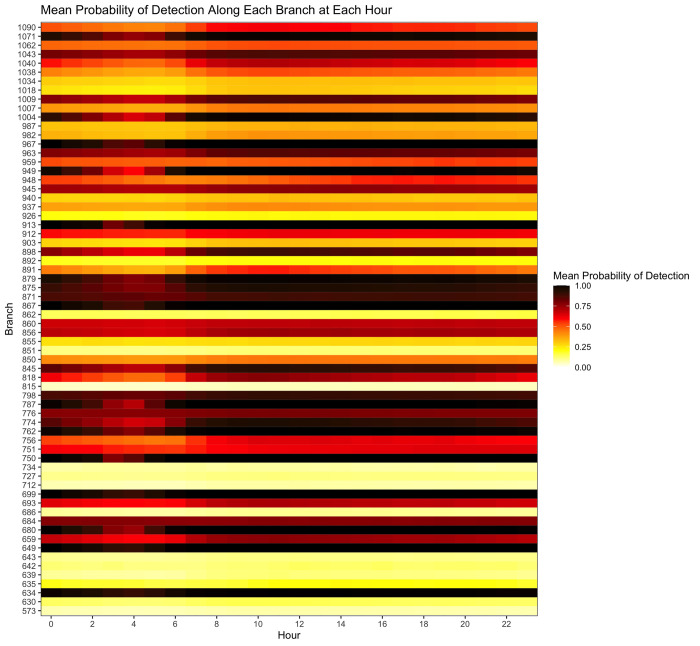
Mean probability of detection along each branch at each hour of the day. For each branch, detection probability is averaged across all 10-meter sections at each hour.

Given the ‘base’ scenario described above (medium prevalence and low flow; Table 2), Fig 3 provides an overview of the mean probability of detection in each branch in the network, by hour. For this modeled scenario, the mean probability of detection across all sections of all branches at all hours was 0.56, with the lowest mean probability occurring at 05:00 (0.48) and the highest mean probability occurring at 12:00 (0.60). Fig 3 does not reveal a noticeable shift in probability of detection over the course of a 24-hour cycle and the range of probabilities over the day is narrow, suggesting that, in this scenario, the time of day does not have a strong influence on the probability of detection.

**Fig 4:**
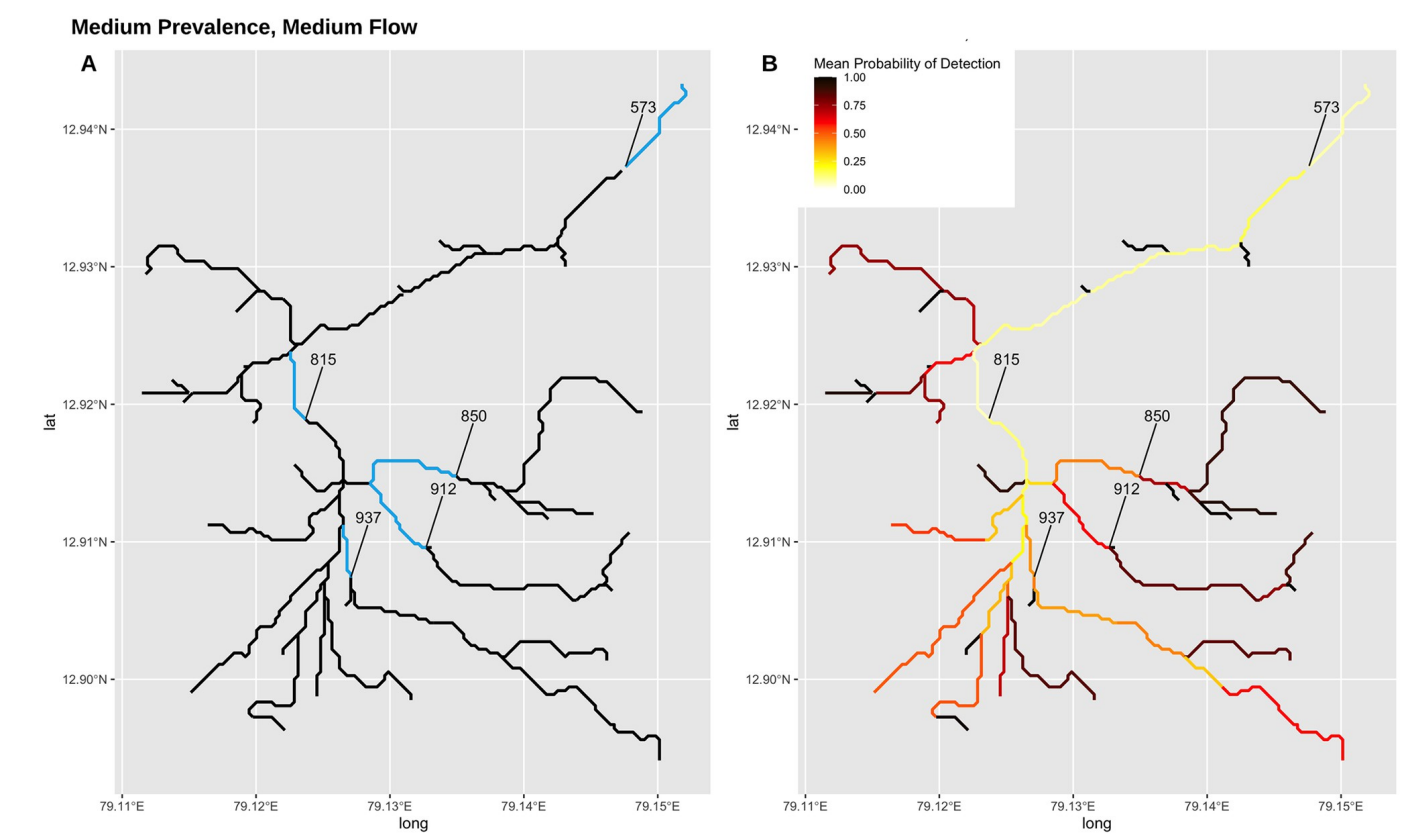
System map with five potential sampling branches. A) Potential sampling locations that emphasize different sampling priorities. B) A visual comparison of the average probability of detection along each sampling branch at 0900. Drainage line data from https://es.world/map/vellore.

Fig 4 provides a mapped overview of the average detection probability along each branch at 09:00. In a scenario with medium prevalence and low flow, dilution and pathogen decay over time appear to play an important role in the downstream concentrations and probability of detection at 09:00 (Fig 4). The highest probabilities of detection are concentrated in upstream branches that do not have ‘parent’ branches, and the lowest probabilities of detection are concentrated in downstream branches with large catchment populations, where larger volumes of wastewater flow and environmental flow converge (Fig 4). When choosing sampling locations, there are multiple competing priorities, including maximizing the total catchment population of all samples, maximizing the probability of detection, minimizing the total number of samples needed to provide an overview of the wastewater network, and the selection of sampling locations that are deemed accessible, suitable, and convenient to a regular sampling schedule. Five candidate sampling branches for this theoretical community include Branches 573, 815, 850, 912, and 937, which represent a range of upstream catchment populations and a range of modeled detection probabilities (Fig 4 and Table 3).

**Table 3:** Five example sampling branches along the theoretical wastewater system.

Branch ID	Branch Population	Cumulative Upstream Population	Branch Length (meters)	Maximum Probability of Detection	Branch Section with Maximum Probability of Detection	Hour of maximum probability of detection
573	2,046	261,917	930	0.16	0	11
815	804	205,278	615	0.10	10	13
850	2,699	23,684	920	0.71	0	12
912	1,511	16,977	760	0.98	0	11
937	1,276	29,193	460	0.56	0	23

Of these five candidate sampling sites, two branches (573 and 815) prioritize capturing large upstream catchment populations and are located downstream of major convergence points in the wastewater network. However, in this model scenario, both branches have relatively low maximum probability of detection, likely due to dilution factors and decay rates as bacteria travel downstream and are continually diluted as wastewater flows converge. Three of these branches (850, 912, and 937) capture smaller cumulative populations, but have much higher estimated detection probabilities. All five of these branches contain sampling sites that have already undergone field-based assessments for site accessibility in a previous study and were deemed suitable and accessible for sampling [10].

**Fig 5:**
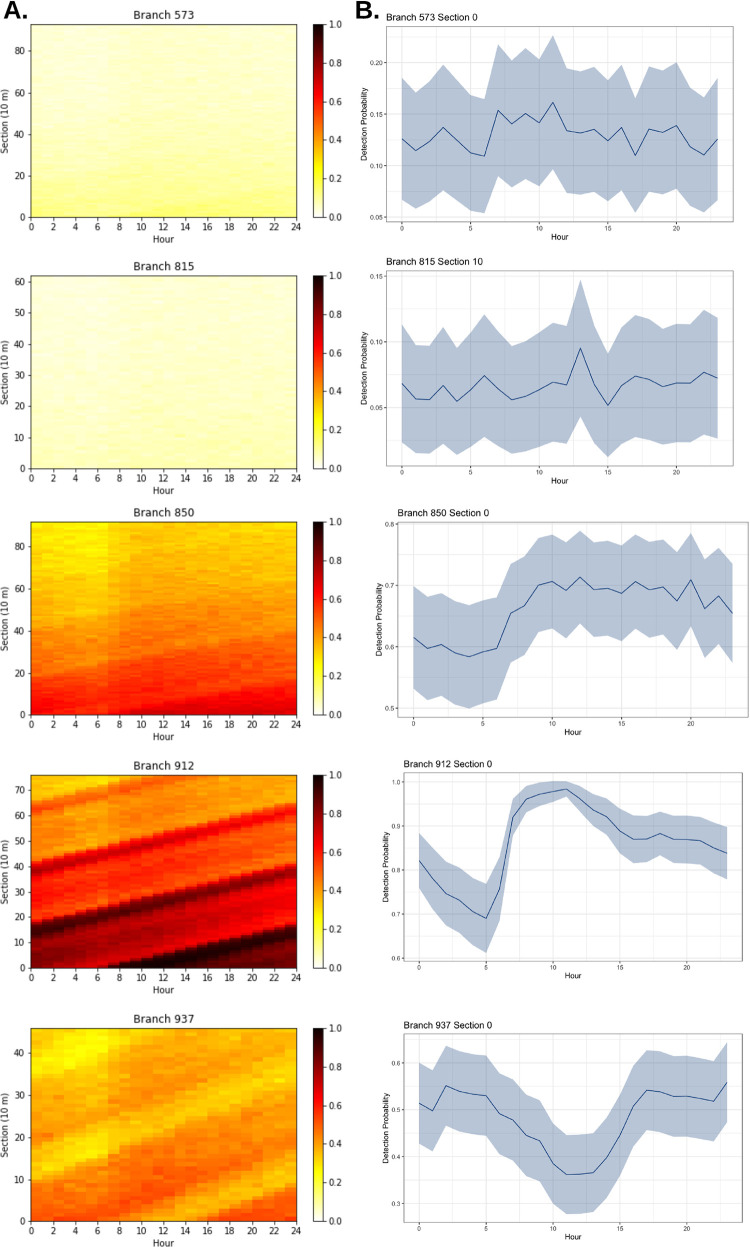
Detection probabilities at five potential sampling branches. A. Probability of detection at each hour and along each section of the five potential sampling branches described in Table 3. B. Probability of detection along the section of the branch with the maximum detection probability, over the course of 24 hours.

The probabilities of detection along each section of each candidate branch over the full 24-hour cycle are plotted in more detail in Fig 5A. In Branches 573 and 815, the overall detection probability across all sections and across all hours is low (less than 0.2). Output from the model suggests which section of the branch and which hour of the day would yield the maximum probability of detection (Branch 573: Section 0 at Hour 11; Branch 815: Section 10 at Hour 13) (Fig 5A and Table 3). Branches 850, 912, and 937, on the other hand, have multiple sections and hours throughout the day where the detection probability is greater than 0.5, and the section of the branch and hour of the day that would yield the maximum probability of detection are identified (here, Section 0 at Hour 12 for Branch 850, Section 0 at Hour 11 for Branch 912, and Section 0 at Hour 23 for Branch 937) (Fig 5A and Table 3). The varying influx of pathogens and wastewater flow into the wastewater network results in the wave patterns apparent in Branches 850, 912, and 937, which visualizes portions of wastewater with higher and lower pathogen concentrations moving downstream along each branch (upwards along the y-axis) over the course of 24-hours (rightwards along the x-axis). An alternative visualization of this wave pattern is shown in S1 Fig, where the peak detection probability occurs over successive hours along the first eleven sections of Branch 912.

Although it is possible to simply calculate the section of each branch and the hour of the day with the highest detection probability, as stated above, sampling sites must also be accessible and sampling hours must fit within a reasonable time frame to avoid logistical barriers like heat, rain, traffic, and the length of time required for sampling [48]. If the section of a branch and the hour of the day with the highest probability of detection is not logistically ideal, Fig 5A suggests that sampling probability is both temporally and spatially autocorrelated within each branch. Temporally, detection probability is moderately autocorrelated (correlations greater than 0.5) for lags up to four hours (S2 Fig). Within each branch, detection probability is moderately spatially autocorrelated along the sections of the branch. Correlations greater than 0.5 occur up to four sections downstream of each section (S2 Fig). This temporal and spatial autocorrelation of the sampling probability along each branch and throughout the day suggest that a sampling field team would be able to utilize sections near the optimal section and sample at hours within four hours of the optimal hour and achieve a probability of detection similar to the maximum modeled detection probability. Branches and sections may also have long stretches of time throughout the day where detection probability is elevated, suggesting that it is not necessary to sample within the 4-hour window of the highest detection probability (Fig 5B). In particular, Branch 850, Section 0 and Branch 912, Section 0 have a peak probability of detection at 12:00 and 11:00, respectively, but the detection probability remains above 50% for the remainder of the day. Branch 937, Section 0 appears to have peak detection probability in the middle of the night (between 23:00 and 02:00). However, detection probabilities greater than 0.5 are also indicated in the late afternoon and early evening (after 16:00) (Fig 5B). This kind of flexibility is helpful in an applied scenario, where a sampling plan must be flexible to logistical considerations.

Finally, 95% confidence intervals in Fig 5B demonstrate the uncertainty associated with detection probabilities at each branch and section (Fig 5B). Uncertainty is higher in the downstream branches, which is likely to do with the higher cumulative uncertainties in flow rates, environmental flow, prevalence, and decay occurring upstream from these branches throughout the entire wastewater network. Branches further upstream, such as 912 and 937, have lower uncertainty, as these branches depend on fewer upstream catchment areas.

Together, the five sampling branches in Table 3 would capture the entire catchment population included in the theoretical study area, but the detection probability is reduced at downstream branches. In this scenario, a more informative ES plan may include more upstream sites that capture as much of the upstream population as possible, while also maintaining a high probability of detection, rather than relying on a downstream convergence branch like 573 to provide a representative sample for the entire catchment population. Alternatively, the sensitivity of sampling at Branch 573 may be increased by increasing the sampling frequency or increasing the number of samples collected at each sampling event (so as to increase total sampling volume). However, these options may be challenging and expensive, and adding one or two upstream sampling sites to replace Branch 573 that will still capture most of the catchment area may be a preferable solution.

### 3.4 Sensitivity analysis

A sensitivity analysis examining three different typhoid prevalence scenarios and four different flow scenarios demonstrated that these two variables have a strong influence on model output. This suggests the possibility that seasonal variability in rainfall and environmental flow and clinical-based estimates of typhoid incidence rates in the community should factor into environmental sampling plans.

#### Prevalence

**Fig 6:**
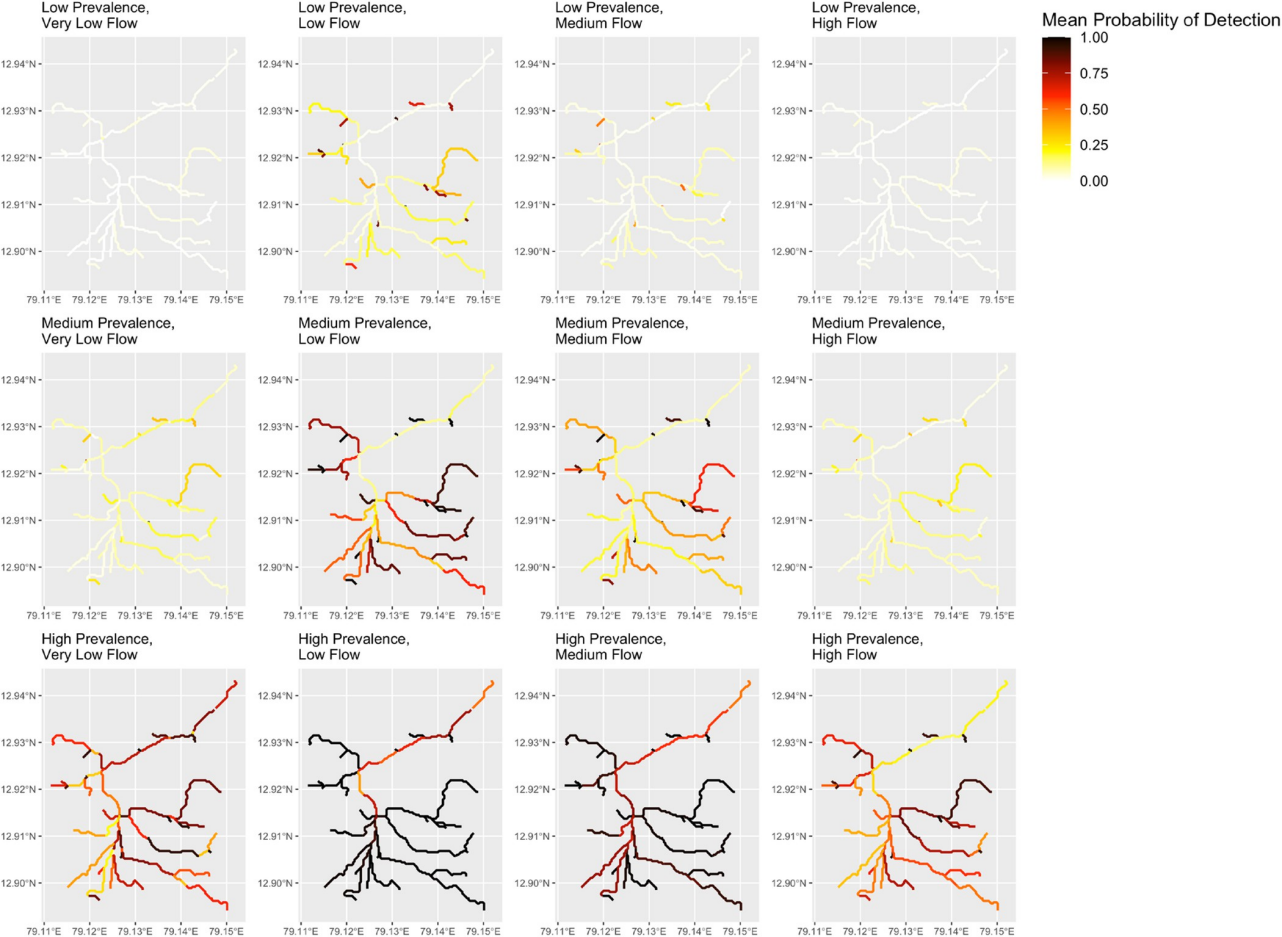
Comparison of system maps across twelve different scenarios described in Table 2. Each subset map provides a visual comparison of the average probability of detection along each branch at 0900. Drainage line data from https://es.world/map/vellore.

Local typhoid prevalence and the prevalence of *S*. typhi shedding (whether symptomatic or asymptomatic) influences the probability of detection throughout the wastewater network (Fig 6). In scenarios with low prevalence, detection probabilities remain low throughout the system, even across different flow scenarios. In the low prevalence, low flow scenario, some branches have higher modeled detection probability, but these are largely limited to upstream branches capturing smaller populations with less total dilution. In these low prevalence scenarios, large environmental surveillance wastewater channels may be unlikely to yield positive detections and sampling further upstream, at shared toilets or the building level, may be warranted, as suggested in Wang et al., 2020 [26]. It is clear from Fig 6 that, when prevalence is low, under any flow scenario, downstream convergence branches with large upstream populations are not ideal sampling locations. If targeted upstream sampling at shared toilets or at the individual street level is not feasible, then an environmental surveillance sampling plan aiming to identify any infections within the catchment population would need to include a greater number of sampling locations, with an emphasis on upstream branches, and an increased sampling frequency and volume, to increase detection probability. In scenarios with medium prevalence, flow rates have a strong effect on detection probability (Fig 6). At low and medium flow rates, some downstream sampling sites with larger catchment areas have detection probabilities greater than 0.5. However, at very low flow and high flow rates, the detection probabilities decrease substantially and, as with low Prevalence, upstream sampling yields higher estimated detection probabilities. In high prevalence scenarios, the selection of sampling location is less important, as the detection probabilities are high throughout the system, particularly in the low flow and medium flow scenarios.

#### Environmental flow

**Fig 7:**
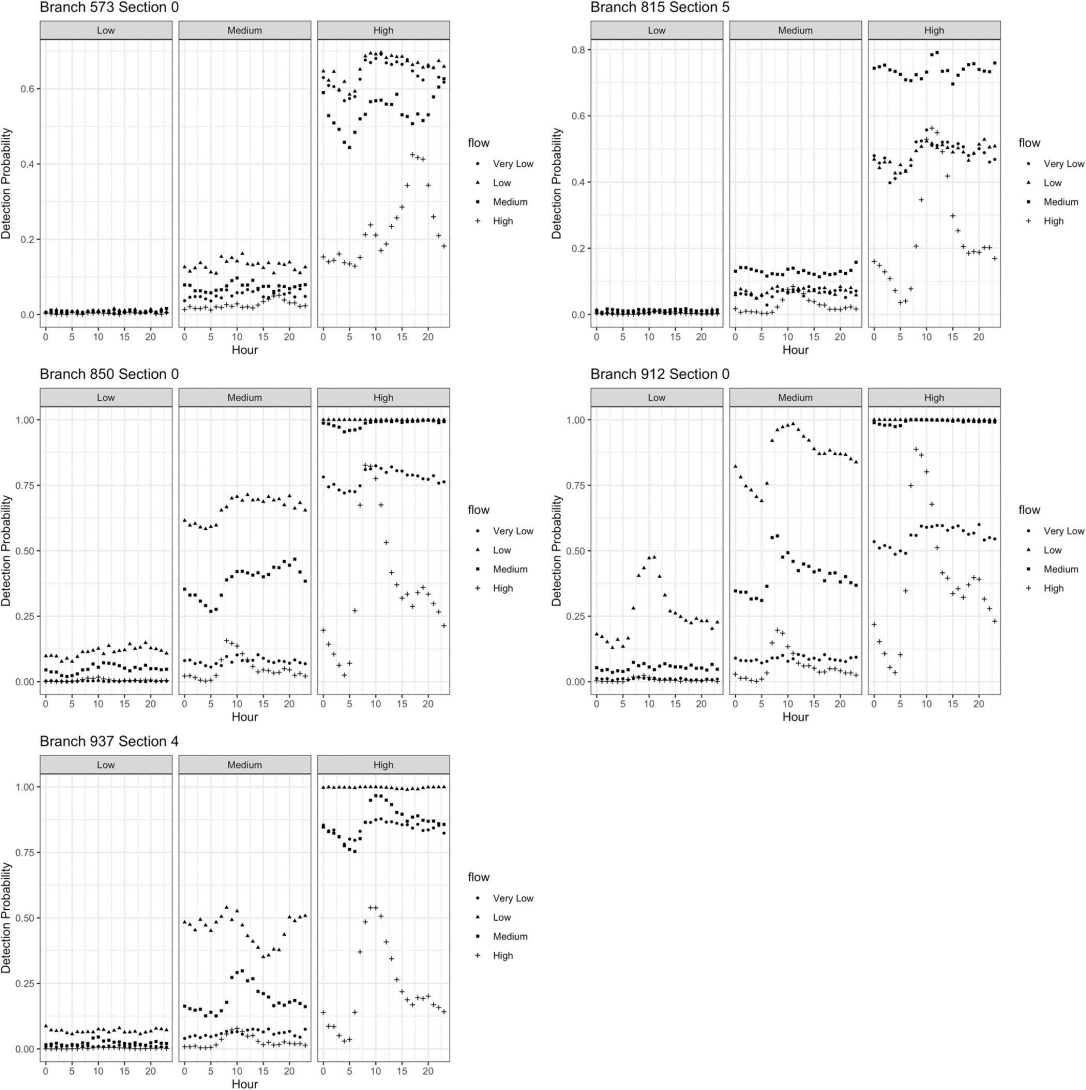
Probability of detection at five sampling points in the wastewater system across 12 different sensitivity analysis scenarios.

The volume of environmental flow also has a strong influence on the probability of detection. High background flow dilutes the pathogens shed into the system, while higher flow also increases the velocity of flow throughout the system, distributing pathogens further downstream and decreasing the residence time (and decay time) in the wastewater network. The same five sampling branches and sections described in Table 3 are plotted in Fig 7 for each sensitivity analysis scenario. As environmental flow (and flow velocity) increases, the temporal dynamics of detection probabilities over the 24-hour hour cycle change. This is particularly clear in the high prevalence and high flow rate scenario, where the probability of detection peaks at each candidate sampling branch at a different hour throughout the day (Fig 7). In all prevalence scenarios, low flow and medium flow yield, on average, the highest detection probabilities across all sampling sites. Both very low flow and high flow rates consistently yield low detection probabilities in the low and medium prevalence scenarios. In the high prevalence scenario, the probability of detection increases for all flow rates, and the probability of detection for the high flow scenario is very time-dependent.

## 4. Discussion

This study presents a dynamic computational model developed to inform environmental surveillance studies in communities that rely on open-channel, informal wastewater networks. A dynamic modeling approach was used to simulate pathogen shedding, fate, transport, dilution, and decay. In open channel networks or environmental streams with high variability in flow, assumptions about minimum and maximum wastewater flow and velocity and pathogen residence in a wastewater network are not applicable [27,50]. This model takes into account environmental flow, which enters open wastewater systems from natural background inflow, infiltration, rainfall, and stormwater runoff and creates an important dilution factor, affecting the probability of detection at any given time and at any given point in the wastewater system. Additionally, this approach considers diurnal variation in wastewater flow rates and pathogen shedding throughout the day to identify optimal sampling times and locations.

An application of this model in a theoretical scenario based on a section of Vellore City, India highlights how model output may be useful in an environmental surveillance study. During sample site selection, model output may be used to identify branches in a wastewater network and sampling times that balance the probability of detection against sampling feasibility (given limiting logistics such as site accessibility, heat, and travel time around a community). In this model application, the effects of pathogen dilution and decay on detection probability were evident in downstream branches. Although downstream branches have larger catchment areas and cover larger proportions of the overall population, they are also likely to have lower sensitivity of detection. As is evident in Fig 4, a negative sampling result at downstream branches such as 573 and 815 does not indicate that there are no infected persons in the wastewater network, and infected persons are likely to be missed by environmental surveillance if these sites are relied upon. In this case, sampling further upstream is more likely to yield positive sampling results. On the other hand, positive sampling results at downstream, highly diluted sites may indicate that local typhoid prevalence is high.

In this theoretical application, the two input variables with the highest degrees of uncertainty and lack of available data are environmental flow and typhoid infection point prevalence. To better understand how these two variables affect model output, sensitivity analyses were run for twelve scenarios, with three different estimates of typhoid prevalence and four estimates of environmental flow. Of the two variables, typhoid prevalence has the stronger effect on model output, where low prevalence (2/100,000) results in low overall detection probability across all flow scenarios, and high prevalence (200/100,000) results in high overall detection probability across all flow scenarios. At medium prevalence (20/100,000), environmental flow has a larger effect on model output: low and medium environmental flows result in higher overall detection probabilities than very low or high environmental flows. This may have implications for environmental surveillance studies conducted in areas with very wet seasons (such as Vellore City, India) and in arid or semi-arid areas with very dry seasons. Environmental sampling results in these areas will need to be interpreted within the context of knowledge about rainfall and environmental flow, as these factors may result in negative sampling results that do not indicate a true lack of cases or transmission.

The results of this sensitivity analysis indicate the potential usefulness of this model in communities where there is no knowledge of typhoid prevalence or transmission. Model set-up requires an assumed community prevalence, but multiple modeling scenarios with a range of community prevalence estimates can easily be run. A comparison of outputs from modeling scenarios with difference prevalence estimates and actual results from ES sampling data may help infer community prevalence estimates–albeit with a very high degree of uncertainty and low precision. Similarly, although prevalence is assigned uniformly across the community in the modeling scenarios presented here, the model setup allows for prevalence to vary by branch. It may also be useful to run modeling scenarios where prevalence is clustered in different areas of the community to determine how output from these scenarios compares to ES sampling data.

The sensitivity analysis also highlights the importance of locally measured environmental flow data when applying this model to different communities and sampling scenarios. Collaboration with hydrologists to accurately determine environmental flow rates for branches would substantially improve model predictions, since dilution is an important driver of detection probability. This kind of cross-collaboration between the fields of environmental microbiology and hydrology has benefitted microbial risk assessment models assessing complex environmental systems [51]. Although measuring the environmental flow in an entire wastewater network may be infeasible, tools designed to estimate streamflow dynamics could be utilized in combination with this model to improve streamflow estimates [52–54]. There are also features of the natural and built environment that are not currently incorporated into this model, which would have a large impact on a wastewater network, such as pump stations, ponds or areas where water accumulates, and local industrial waste flows. It would be useful to add optional pump stations at known locations in order to reflect how those would change the rate of flow in a system and as potential sampling points.

Multiple previous studies have produced models to optimize sampling site selection for environmental surveillance [21–24]. These modeling studies took a variety of approaches, including optimization algorithms aimed at maximizing catchment population while minimizing the number of required sampling points [22,23] and adaptive optimization algorithms that iteratively improved upon detection sensitivity in response to rounds of sampling results, with the goal of identifying disease clusters or hot spots ([25,26]). A typhoid-specific environmental sampling model published in 2020 also incorporated shedding dynamics and pathogen fate and transport [26]. This model builds on previous work by modeling shedding dynamics, pathogen fate, transport and decay in an open channel system while also incorporating flow dynamics, dilution effects, varying flow velocity, and diurnal variation in both shedding and flow volume. This study also highlights the importance of modeling pathogen dilution and investigates how changes in flow rates affect detection probabilities.

This study does not propose an automated algorithm or decision tree to build an ES sampling strategy as the aim was not to improve upon existing and high-performing algorithms for sampling plan development. One possible future direction of this work would be to adapt site allocation algorithms from previous modeling studies to use in conjunction with this model to improve sampling plans over time and adapt sampling plans to different flow conditions (resulting from seasonal rain patterns) and different prevalence estimates. This model was developed to inform the development of sampling plans and to aid in the interpretation of sampling results. ES results can be difficult to interpret, and both positive and negative samples raise further questions. Does a positive result mean that a very large number of infected persons are shedding or does it indicate a small cluster of people shedding? Does a negative result mean that there are no infected persons shedding in the community, or is it possible that there are infected person in the community but the shed pathogens are too diluted to be detected in wastewater? These questions are essential for public health interpretation of ES data, but they are also extremely difficult to answer, due to the high degree of uncertainty in environmental and wastewater surveillance [17]. While this relatively simple model will certainly not provide definitive answers to these questions, it may–when parameterized with locally relevant data–provide some helpful insight into the conditions that may produce positive or negative results at different sampling points and times of day.

An important future step to improve upon this analysis will be to run this model (using a wider range of prevalence estimates and with more accurate environmental flow estimates) in a community with an existing ES program for typhoid fever and to compare modeling output to sampling results. This will help to validate the model and identify areas for improvement, and it would provide a real-world scenario in which to determine whether the model output is useful in the interpretation of ES results. An ES study is underway in Vellore City, India, in a part of the city that partially overlaps with the theoretical system presented here, and the sampling results from this study could provide an ideal dataset for this purpose [11].

## 5. Conclusion

The model presented here provides the built-in flexibility to utilize both locally obtained and published data to model detection probabilities at different sampling locations and times. As is the case with any modeling exercise, the model output is closely tied to the representativeness and accuracy of the input data. However, despite challenges in producing reliable absolute estimates of detection probability, relative estimates provide utility in determining when, where, and how sampling should take place. Model estimates are also useful in the interpretation of sampling results. When designing an environmental sampling plan–particularly in a non-centralized wastewater network—it may not be clear how sensitive of a laboratory method is required, how many samples to collect, what volume to collect, when to collect samples, and which sampling locations to prioritize. This model provides a framework to begin answering these questions and a foundation to build upon to produce more specific models on a community-by-community basis. The flexibility of this model has been prioritized in its development, and it has been designed to be applied, adjusted, and improved to support environmental surveillance efforts by public health practitioners, researchers, and utility operators.

## Supporting information

S1 Text Model Input Parameters and Assumptions. Fig A. Probability of shedding, given days since infection. Fig B. Estimated shedding load. Table C: Diurnal Variation in Defecation Rates. Fig D. Mean diurnal variation in wastewater flow across three study locations.
(DOCX)

S2 Text Model Procedure. Fig A: Diagram of the pathogen and flow loading processes.
(DOCX)

S1 Fig Probability of detection at each hour along the first eleven sections of Branch 912. (TIFF)

S2 Fig Temporal and spatial autocorrelation in detection probability along five branches in the wastewater system in the ‘Medium Prevalence, Low Flow’ scenario. (TIFF)
