# Supplementary material for: Development of a computational model to inform environmental surveillance sampling plans for Salmonella enterica serovar Typhi in wastewater
Source: PLoS Negl Trop Dis. 2024 Mar 29;18(3):e0011468. doi: 10.1371/journal.pntd.0011468 (PMC11020695; doi:10.1371/journal.pntd.0011468)
Supplement: S1 Text — Fig A. Probability of shedding, given days since infection. Fig B. Estimated shedding load. Table C: Diurnal Variation in Defecation Rates. Fig D. Mean diurnal variation in wastewater flow across three study locations. (DOCX) [file pntd.0011468.s001.docx]

# **S1 Text.** **Model Input Parameters and Assumptions.**

Input parameters required for the model were gathered from available literature, where possible. Parameter distributions and sources are described in the main text in Table 1.

**Prevalence of infection.** The prevalence of infection for *S.* Typhi is not well-characterized and systemic surveillance for typhoid fever has been limited in many countries [1]. A recently published Surveillance for Enteric Fever in India (SEFI) study conducted active blood culture surveillance of children under 15 years of age in Vellore, India from 2017 to 2020, estimating a disease incidence in this age group of 1,173 cases per 100,000 child-years and a mean fever duration of 9 days. This incidence rate was adjusted for the relatively low sensitivity of blood culture (60%) [1,2], but it did not adjust for prior antibiotic use, days since fever onset, or include persons greater than 15 years of age. Given the uncertainty of translating this incidence rate to a population point prevalence of all infected symptomatic and asymptomatic individuals that may be shedding *S*. Typhi into the wastewater, a point prevalence of 20 infections per 100,000 persons was used as a ‘base’ case or ‘medium’ prevalence in this study. Sensitivity analyses were conducted to simulate scenarios with point prevalence estimates ten time lower (2 infections per 100,000 persons) and ten times higher (200 infections per 100,000 persons) than this point prevalence.

**Intermittent shedding.** Among those infected with *S.* Typhi, there is evidence that fecal shedding occurs intermittently in the days and weeks following the initial infection [3]. The duration of shedding and the window of time during which intermittent shedding may occur is not well defined, varies between individuals, and may continue for more than 30 days and, more rarely, for more than 180 days [3–5]. Furthermore, a small proportion of infected individuals become chronic carriers [5,6]. In order to model intermittent shedding, the probability of shedding, given days since infection, was inferred from data from 6 human challenge studies conducted between 2011 and 2017, including 331 participants challenged with *S.* Typhi [3]. In these studies, antibiotics were initiated on day 14 for all subjects, regardless of symptoms, and no further stool samples were collected. From these challenge studies, it is not possible to infer the probability of shedding after day 14. For this analysis, therefore, Typhoid infections were randomly assigned ‘Day since infection’ up to 14 days (with equal weights), and the probability of shedding was assigned according to the probability of shedding shown in Fig A. Chronic carriers and chronic shedding were not considered.

**Fig A.** **Probability of shedding, given days since infection.**


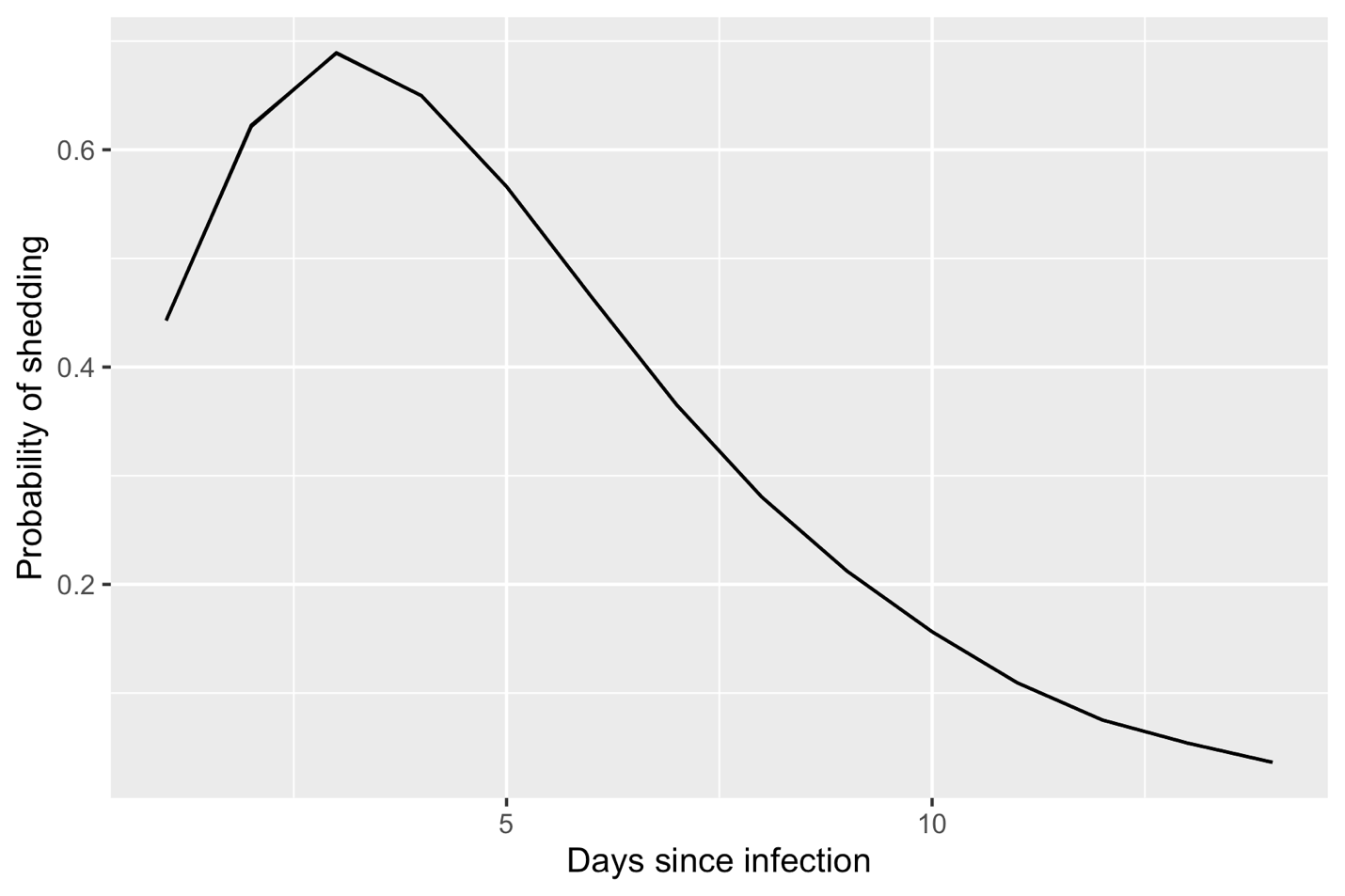


**Shedding load.** Expert opinion was sought to assign a shedding load for *S.* Typhi, which was simulated as a lognormal distribution (Fig B).

## **Fig B.** **Estimated shedding load.**


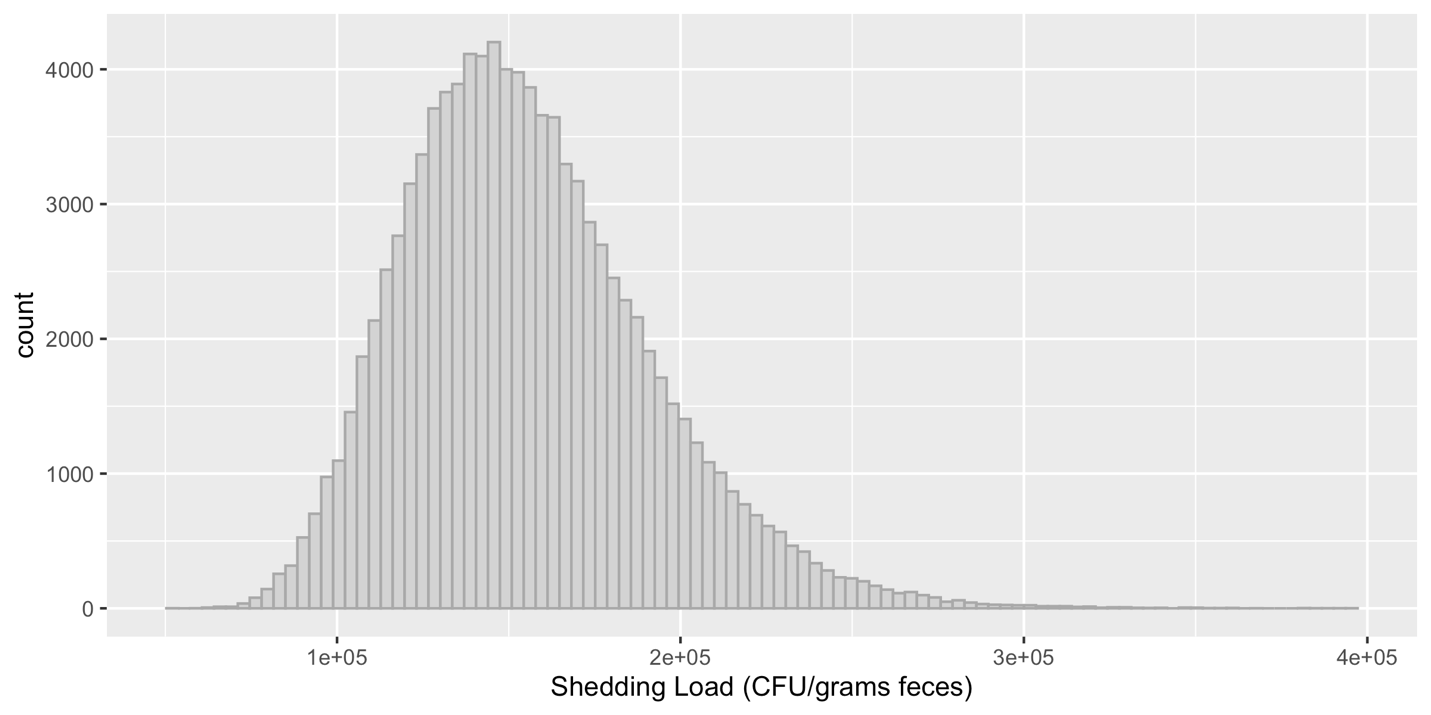


**Fecal output per day and 24-hour fluctuations in output.** An estimate for the daily fecal output per person was obtained from a 2015 study reviewing the generation rate of the solid and liquid fractions of human excreta in developing countries [7]. The average daily wet fecal weight of fecal output per person from low income countries was estimated to be a non-skewed normal distribution with a mean of 243 grams/person-day (σ = 130), which was the distribution used for this analysis [7].

To simulate 24-hour fluctuations in fecal output per day, a 1992 study tracking the timing of defection in 838 adult men and 1059 adult women in East Bristol was used to produce an approximate estimation for diurnal variation in fecal output (Table C). This data from this study population is unlikely to be directly translatable to the population of Vellore City, India, as defection timing and frequency is likely to vary by diet and age group. In particular, children under the age of 15 are at the highest risk of developing *S.* Typhi infection [1], and the diets of adults in Britain are different from the diets of children in Southern India. However, to our knowledge, no other published study has conducted surveys for defecation rates and frequency over the course of a 24-hour day.

##

## **Table C: Diurnal Variation in Defecation Rates**

| **Hour** | **% of Total Daily Defecation** | **Hour** | **% of Total Daily Defecation** |
| --- | --- | --- | --- |
| 0^*^ | 2.0 | 12 | 2.5 |
| 1 | 0.3 | 13 | 3.5 |
| 2 | 0.3 | 14 | 3.3 |
| 3 | 0.1 | 15 | 2.5 |
| 4 | 0.3 | 16 | 3.3 |
| 5 | 1.3 | 17 | 2.3 |
| 6 | 6.0 | 18 | 4.3 |
| 7 | 19.5 | 19 | 4.0 |
| 8 | 17.5 | 20 | 2.5 |
| 9 | 10.3 | 21 | 1.8 |
| 10 | 7.8 | 22 | 2.0 |
| 11 | 5.5 | 23 | 1.8 |

* Hour 0 corresponds to midnight (0000)

**Wastewater flow per capita per day and 24-hour fluctuations in output.** Data on wastewater flow per capita were obtained from a 2022 study investigating wastewater generation in India [8]. The per capita sewage production estimate from Tamil Nadu was used for this study (100 liters / day-person) and a normal distribution was inferred (N(μ = 100, σ = 50)).

To simulate 24-hour fluctuations in wastewater generation, no published sewer hydrograph data from India was found. To estimate diurnal wastewater flow variation in Vellore City, India, 24-hour hydrograph data from three other studies were combined and mean flow values across these studies were used. Studies included diurnal wastewater flow fluctuations in New Cairo, Egypt, nine different communities across a range of socioeconomic categories within Belo Horizonte, Brazil, and a pumping station in Tulkarem City, Palestine [9–11]. These three locations and the represented populations vary in geography, culture, climate, and socioeconomic factors and the study time periods ranged widely, from 1996 to 2014. However, the observed diurnal flow pattern followed a similar trajectory across all three studies, and the mean of these three study estimates for flow throughout that 24-hour day was used (Fig D).

## **Fig D.** **Mean diurnal variation in wastewater flow across three study locations.**


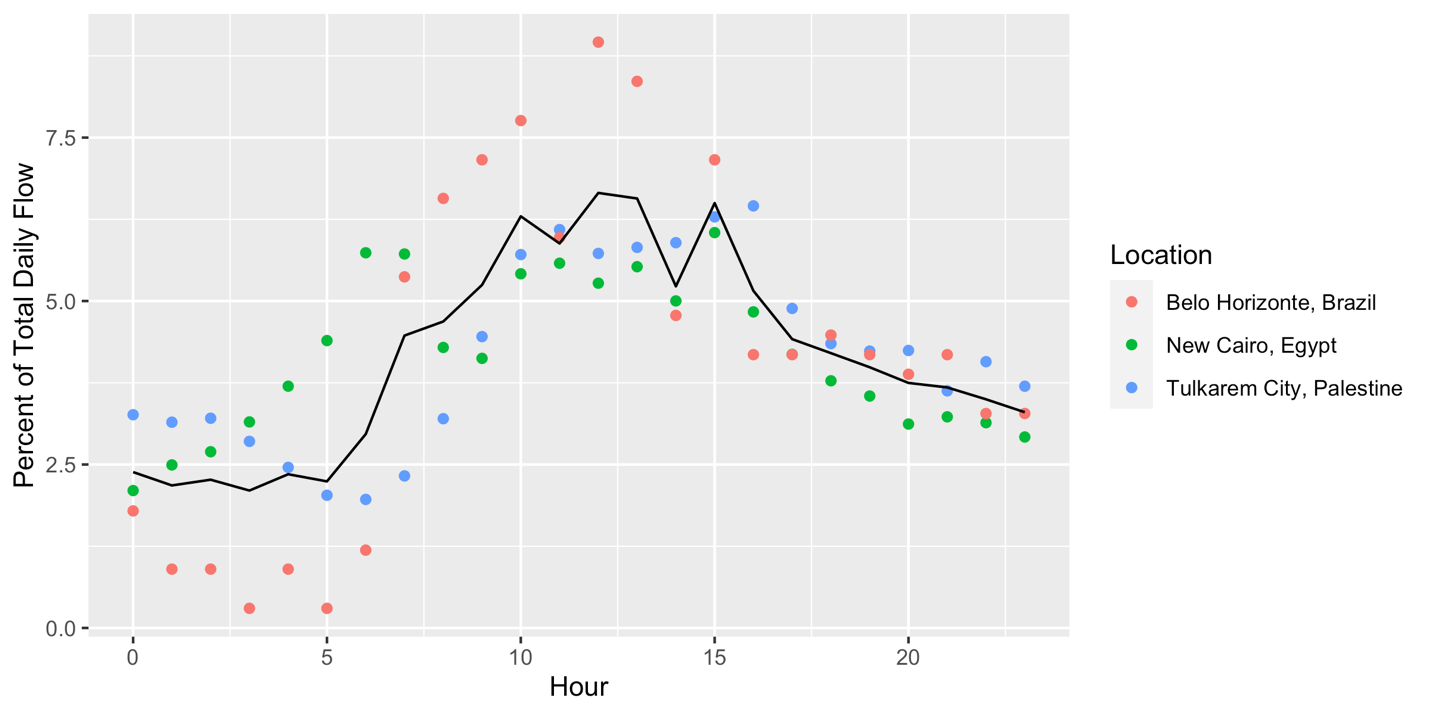


**Environmental Flow.** Very little published flow data are available for open channel wastewater flows in urban or rural areas of India. The ‘Low’ flow estimate in this analysis is gathered from a 2008 study measuring the open channel flow over the course of a day in two villages in Punjab, India [12] (Table 1). These two rural locations lack comparability with an urban environment in southern India like Vellore City in terms of region, seasonal weather patterns, catchment population size, and population density. Given a lack of more comparable published data on open channel wastewater flow rates, the mean hourly flow rate for these two villages was used in this study to describe a ‘Low’ flow scenario in Vellore City, India (given the much smaller population represented in these villages as compared to Vellore City). Three additional flow scenarios were included. ‘Very Low’ flow was defined as a mean flow rate ten times lower than the rates described in Singh, 2017, in order to evaluate a very dry scenario. ‘Medium’ and ‘High’ flow scenarios were defined as mean flow rates ten times and 100 times higher than ‘Low Flow’ to evaluate higher flow scenarios and dilution effects that are more likely to exist in a large, densely populated urban area and during rainier, wetter scenarios (Table 1).

**Pathogen decay rate.** For this study, pathogen decay rates were defined as the rate of decay of live *Salmonella* bacteria in wastewaters or environmental waters. The decay rate of live pathogens (in this case, bacteria) was chosen because multiple methods utilized for typhoid ES utilize an enrichment step, requiring the presence of live bacteria [13,14]. However, different decay rates could be utilized. For example, if a method did not require live bacteria and enumerated the number of gene copies in a wastewater sample, then decay rates of genetic material in wastewater would be more applicable than the decay rates of live bacteria. Only one study was found investigating the survival of *S.* typhi bacteria in environmental water and this study only tested groundwater and pond water and did not examine wastewater [15]. Another study examined the survival of *Salmonella enterica* in general in dairy lagoon wastewater, which may be a closer approximation to human wastewater than pond or groundwater, but is still imperfect and not specific to *S.* typhi bacteria [16]. More data is needed to understand the decay rates of *S.* Typhi in wastewater. For this study, the decay rates reported in these two studies were randomly sampled with equal weights for each model simulation.

**S****upplemental References**

1. John J, Bavdekar A, Rongsen-Chandola T, Dutta S, Gupta M, Kanungo S, et al. Burden of Typhoid and Paratyphoid Fever in India. N Engl J Med. 2023 Apr 20;388(16):1491–500.

2. Antillon M, Saad NJ, Baker S, Pollard AJ, Pitzer VE. The Relationship Between Blood Sample Volume and Diagnostic Sensitivity of Blood Culture for Typhoid and Paratyphoid Fever: A Systematic Review and Meta-Analysis. J Infect Dis. 2018 Nov 10;218(suppl_4):S255–67.

3. Gibani MM, Voysey M, Jin C, Jones C, Thomaides-Brears H, Jones E, et al. The Impact of Vaccination and Prior Exposure on Stool Shedding of Salmonella Typhi and Salmonella Paratyphi in 6 Controlled Human Infection Studies. Clin Infect Dis. 2019 Apr 8;68(8):1265–73.

4. Khanam F, Darton TC, Meiring JE, Kumer Sarker P, Kumar Biswas P, Bhuiyan MAI, et al. Salmonella Typhi Stool Shedding by Patients With Enteric Fever and Asymptomatic Chronic Carriers in an Endemic Urban Setting. J Infect Dis. 2021 Dec 15;224(Supplement_7):S759–63.

5. Gauld JS, Hu H, Klein DJ, Levine MM. Typhoid fever in Santiago, Chile: Insights from a mathematical model utilizing venerable archived data from a successful disease control program. PLoS Negl Trop Dis. 2018 Sep 6;12(9):e0006759.

6. Gopinath S, Carden S, Monack D. Shedding light on Salmonella carriers. Trends Microbiol. 2012 Jul 1;20(7):320–7.

7. Rose C, Parker A, Jefferson B, Cartmell E. The Characterization of Feces and Urine: A Review of the Literature to Inform Advanced Treatment Technology. Crit Rev Environ Sci Technol. 2015 Sep 2;45(17):1827–79.

8. Minhas PS, Saha JK, Dotaniya ML, Sarkar A, Saha M. Wastewater irrigation in India: Current status, impacts and response options. Sci Total Environ. 2022 Feb 20;808:152001.

9. Imam EH, Elnakar HY. Design flow factors for sewerage systems in small arid communities. J Adv Res. 2014 Sep 1;5(5):537–42.

10. Shaheen HQ. Municipal wastewater characteristics at Irtah pumping station in the Tulkarem city. Water Sci Technol. 2000 Jul 1;42(1–2):337–40.

11. Campos HM, von Sperling M. Estimation of domestic wastewater characteristics in a developing country based on socio-economic variables. Water Sci Technol. 1996 Jan 1;34(3):71–7.

12. Singh S. Determination of Flow Rate of Wastewater Using V-Notch Weir in Villages of Punjab (India). Int J Recent Eng Res Dev IJRERD. 2017 Apr;2(4):81–6.

13. Zhou N, Ong A, Fagnant-Sperati C, Harrison J, Kossik A, Beck N, et al. Evaluation of Sampling and Concentration Methods for Salmonella enterica Serovar Typhi Detection from Wastewater. Am J Trop Med Hyg. 2023 Mar 1;108(3):482–91.

14. Uzzell CB, Troman CM, Rigby J, Mohan VR, John J, Abraham D, et al. Environmental surveillance for Salmonella Typhi as a tool to estimate the incidence of typhoid fever in low-income populations. Wellcome Open Res [Internet]. 2023 Jan 6 [cited 2023 Oct 6]; Available from: https://wellcomeopenresearch.org/articles/8-9/v1

15. Cho JC, Kim SJ. Viable, but non-culturable, state of a green fluorescence protein-tagged environmental isolate of Salmonella typhi in groundwater and pond water. FEMS Microbiol Lett. 1999 Jan 1;170(1):257–64.

16. Ravva SV, Sarreal CZ. Survival of Salmonella enterica in aerated and nonaerated wastewaters from dairy lagoons. Int J Environ Res Public Health. 2014 Oct 29;11(11):11249–60.
