# Supplementary material for: Development of a computational model to inform environmental surveillance sampling plans for Salmonella enterica serovar Typhi in wastewater
Source: PLoS Negl Trop Dis. 2024 Mar 29;18(3):e0011468. doi: 10.1371/journal.pntd.0011468 (PMC11020695; doi:10.1371/journal.pntd.0011468)
Supplement: S2 Text — Fig A: Diagram of the pathogen and flow loading processes. (DOCX) [file pntd.0011468.s002.docx]

**S2 Text. Model Procedure.**

At the beginning of each simulation, each branch is ‘empty,’ containing zero pathogens and zero liters of water. Branches are automatically subdivided into decameter-long sections, and model updates occur for each section of each branch. These updates are performed hourly for both pathogen loading and wastewater flow loading. Fig A1 visualizes the overall loading process for both pathogens and flow for Branch 684, Section 2 of the case study wastewater drainage system described in the main text (Fig 1). Fig A2 provides a more detailed diagram of the cumulative loading estimation for Branch 684, Section 2.

# **Fig A: Diagram of the pathogen and flow loading processes.**


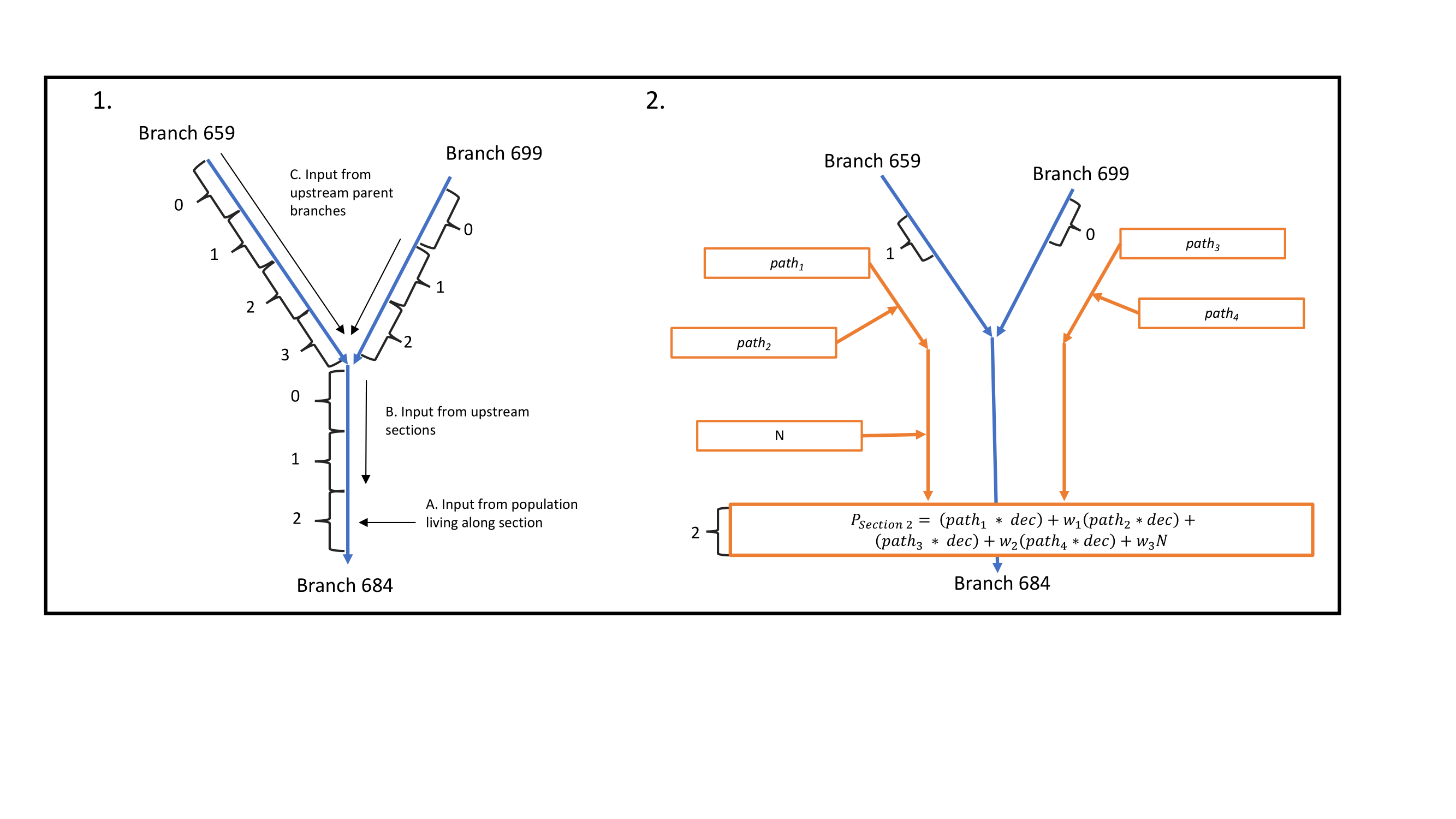


**Model Loading**

The number of pathogens (*N*) entering the system at each section of each branch of the system is calculated according to Eq. [1].

$N = {percent}_{def} * [\sum_{i=1}^{i=p} {(d}_{i}*{sh}_{i})]]$ **[1]**

where ${percent}_{def}$ is the percent of total daily defecation occurring at the specified hour, *p* is the number of persons infected with *S.* typhi along the branch (# of persons), *d_i_* is the defecation rate of each infected person (*i*) (g/day*person), and sh_i_ is the shedding rate of each infected person (*i*) (CFU/gram)*.*

The distance travelled (*D*) by the pathogens and by water flow is calculated according to Eq. [2].

$D = t * v$ **[2]**

where *t* is time since last update (1 hour) and *v* is velocity of flow in the relevant branch (extrapolated from the flow velocity (meters/hour) to sections/hour).

The estimated number of pathogens (*P*) in a section of a branch along the system at a given time is calculated by Eq. [3].

$P = \left( path * dec \right)+ N$ **[3]**

where *path* is the number of pathogens feeding into current section from upstream sections (Arrow B in in Fig A1), *dec* is decay rate (hour^-1^), and *N* is the number of pathogens entering the system at that hour from the local population, calculated in Eq. 1 (Arrow A in in Fig A1). If the upstream pathogens feeding into the current section of a branch have travelled farther than the length of that branch (*D,* calculated according to Eq. [2]), the model recursively calculates the cumulative pathogen load from upstream parent branches, given the estimated velocity of each branch and the total distance travelled since the previous hour (Arrow C in in Fig A1).

This loading process is visualized in more detail in Fig A2, which gives an example of pathogen loading of Branch 684, Section 2 at Hour 9. For modeling purposes, the wastewater network is conceptualized as a series of ‘compartments’, containing pathogens (and flow volume) that travel at varying velocities as they flow downstream through the system. Given the velocity of the water traveling along each branch, the model estimates that the ‘compartments’ present in Branch 659, Section 1 at Hour 8 (*path_1_*) and in Branch 699, Section 0 at Hour 8 (*path_3_*), will travel to Branch 684, Section 2 during one hour of flow. Over this hour, more pathogen loading may occur along Branches 659, 699, and 684 (*path_2_, path_4_*, and N), depending on the number of infections shedding along each branch. The pathogen loading occurring during one hour from each branch is weighted according to the proportion of the hour that the ‘compartments’ theoretically spent in each branch while flowing downstream to Branch 684, Section 2 (*w_1_*, *w_2_* and *w_3_*), given the flow velocity of each branch. *P_Section2_*  is calculated from the cumulative pathogen load from upstream compartments traveling downstream from parent branches (*path_1_* and *path_3_,* multiplied by the pathogen decay rate), the additional pathogen loading over the course of the hour from both upstream branches (*path_2_* and *path_4_*_,_ weighted by the proportion of the hour spent in each parent branch), and the pathogen loading over the course of the hour from Branch 684 (N, weighted by the proportion of the hour spent in the present branch).

**Flow Loading**

To calculate the total water volume in each section of each branch, the model calculates the input of wastewater flow and environmental flow at each hour of the day. The estimated flow volume (W) in the system at a given section of a given branch is calculated according to Eq. [4].

$W = inf +({percent}_{flow} * fl*pop) + env$ **[4]**

where *inf* (liters) is the influx of flow from upstream sections flowing into the current section (Arrow B in Fig A1), *fl* (liters/person-day) is the wastewater flow generated per person over the course of a day, *pop* (person) is the estimated population living along that section of the branch, ${percent}_{flow}$is the percent of total daily wastewater flow occurring at the specified hour, and *env* is the environmental background flow (liters/hour). Arrow A in Fig A1 represents the combined wastewater and environmental flow in the system at the current section of the current branch. Similar to the pathogen loading, if the upstream flow entering the current section of a branch has travelled farther than the length of that branch since the previous hour, the model recursively calculates the cumulative flow loading from all upstream parent branches (Arrow C in Fig A1). This cumulative loading process follows the same procedure described in Fig A2 for pathogen loading, where *W_Section2_*  is equal to the cumulative flow loading from all upstream sections and branches over the course of the hour (although there is no decay rate included for flow).

**Pathogen Concentration**

Given the pathogen load estimate (P) in Eq [3] and the water volume estimate (W) in Eq [4] at each section of each branch, the model calculates and stores the estimated concentration (CFU/liter) of *S.* Typhi (C) at each section of each branch at the given hour.

$C = \frac{P}{W}$ **[5]**

**Probability of Detection**

The user provides a value for the probability of a positive result (*P*), given some known number of *S.* Typhi bacteria (*k*) in 1 liter of wastewater. The probability of a positive result should be estimated from laboratory data or published literature about the ES method of choice. This probability (*P*) is then used to calculate a Beta parameter, which represents the sensitivity of the method; the Beta parameter is used to calculate the probability of a positive result in a sample of wastewater with specified estimated concentration of pathogens.

$\beta= \frac{-ln(1-P)}{k}$ **[6]**

For each section of each branch, the estimated concentration (*C)* of pathogens per liter and a user-specified sampling volume (*S*) (liters) are multiplied to define the shape parameter for a Poisson distribution (*p(b)*) defining the pathogen counts in a given sample volume (Eq. [7]).

$p(b) =$Poisson$\left[ b; S * C \right]$ **[7]**

The Poisson distribution (Eq. [7]) is randomly sampled for the number of pathogens captured in a single sampling volume of *S*. The number of pathogens (*b*) in a collected sample is then used to calculate the probability of a positive laboratory result for *S.* Typhi, via Eq. [8], given the previously defined parameter ($\beta)$.

$p(+) = 1 - exp(-\beta* b)$ **[8]**

p(+) is the probability of a positive result, $\beta$ is the laboratory method’s sensitivity parameter, as calculated in Eq. [6], and *b* is the randomly sampled number of bacteria in the sample. This process is repeated with 1,000 random samples from the Poisson distribution. The mean detection probability (and standard deviation) across all random samples is returned for each section of each branch at each hour. The equations used to estimate the probability of detection (Eq [6], Eq [7], and Eq[8]) were obtained from Ranta et al., 2001 [1].

**Supplemental References**

1. Ranta J, Hovi T, Arjas E. Poliovirus surveillance by examining sewage water specimens: studies on detection probability using simulation models. Risk Anal Off Publ Soc Risk Anal. 2001 Dec;21(6):1087–96.
