# Supplementary figures and images for: Development of a computational model to inform environmental surveillance sampling plans for Salmonella enterica serovar Typhi in wastewater
Source: PLoS Negl Trop Dis. 2024 Mar 29;18(3):e0011468. doi: 10.1371/journal.pntd.0011468 (PMC11020695; doi:10.1371/journal.pntd.0011468)

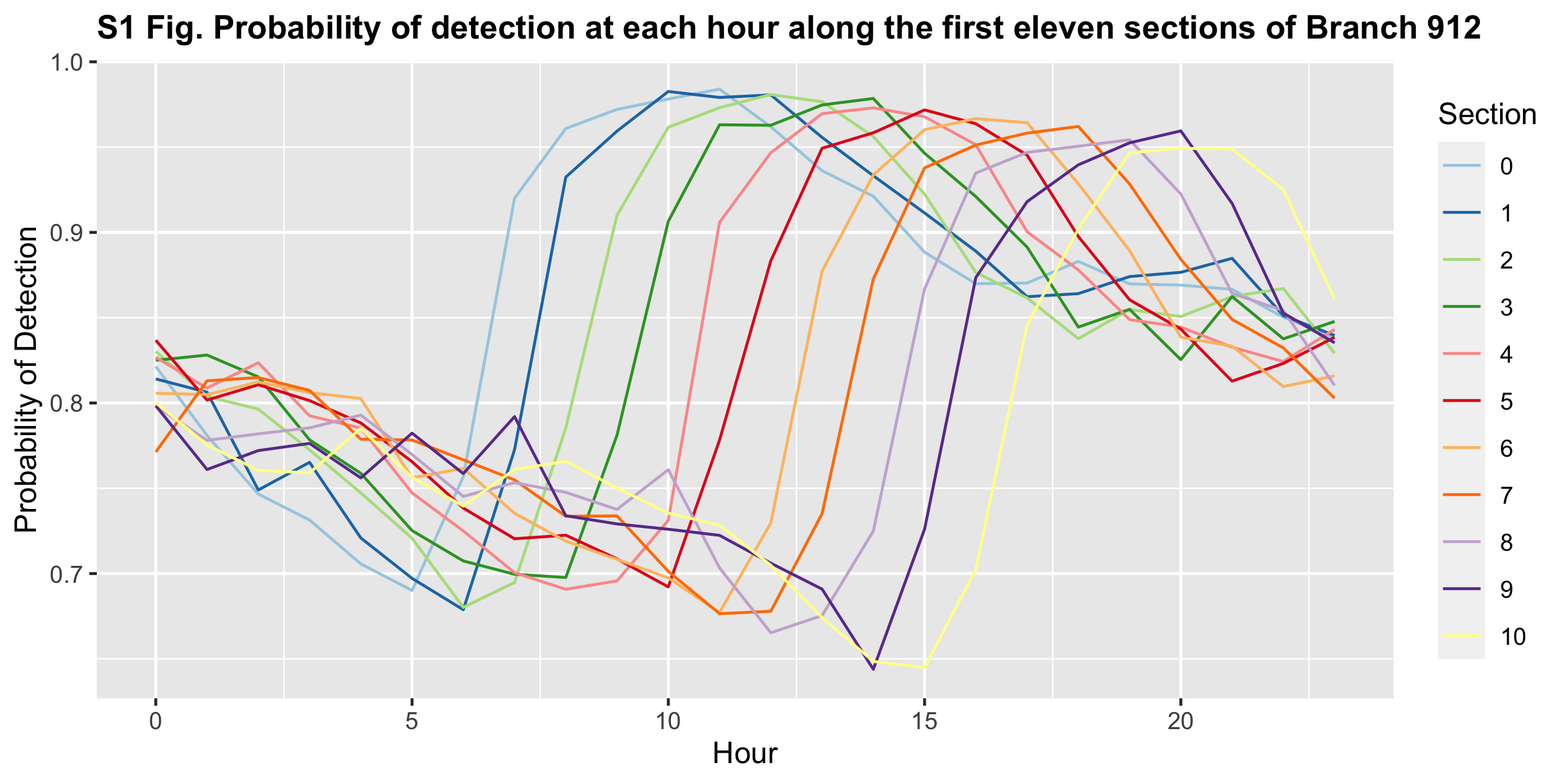

Supplement: S1 Fig — (TIFF) [file pntd.0011468.s003.tiff]

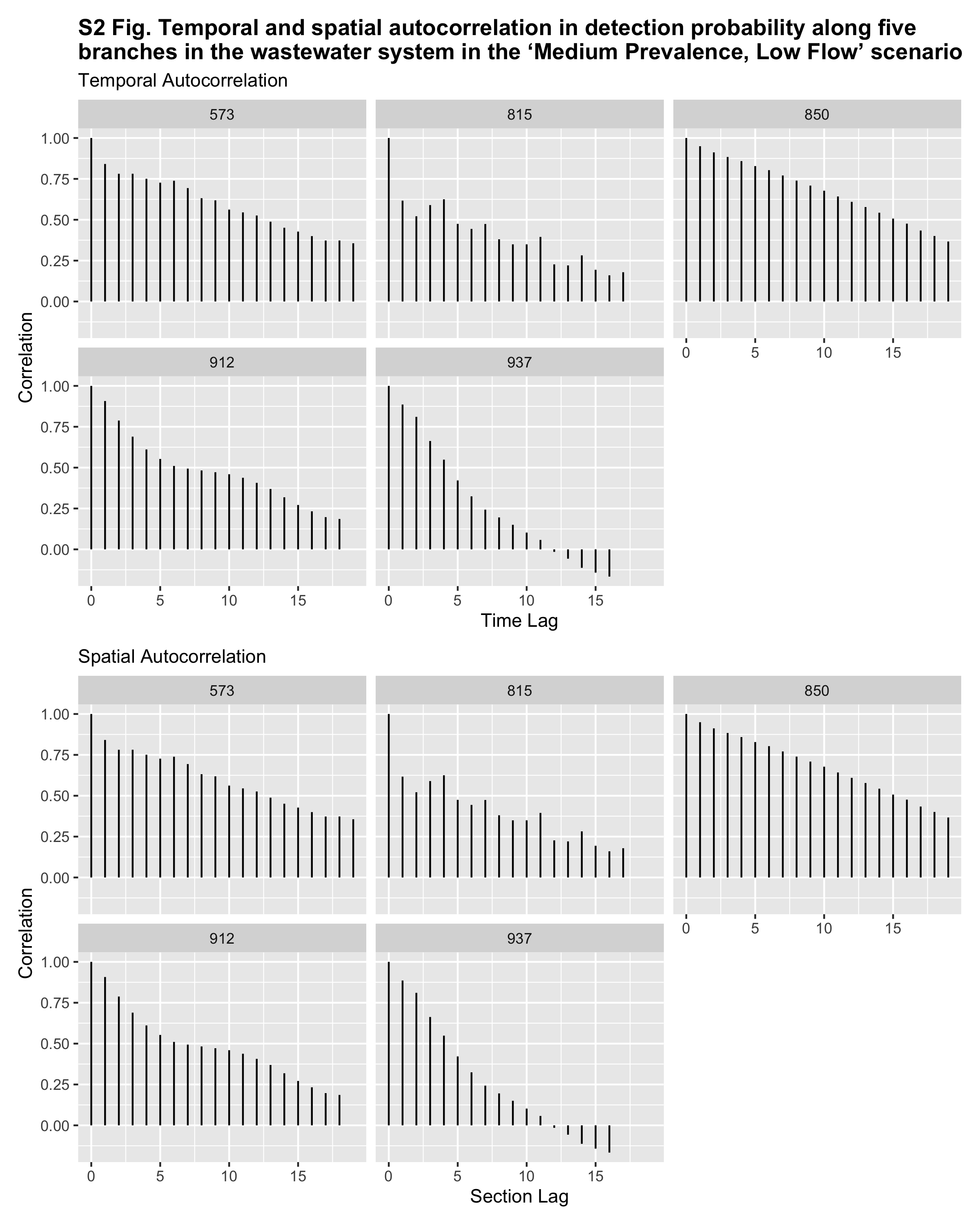

Supplement: S2 Fig — (TIFF) [file pntd.0011468.s004.tiff]
